# Quantifying the transport of biologics across intestinal barrier models in real-time by fluorescent imaging

**DOI:** 10.3389/fbioe.2022.965200

**Published:** 2022-09-09

**Authors:** Arjen Weller, Morten B. Hansen, Rodolphe Marie, Adam C. Hundahl, Casper Hempel, Paul J. Kempen, Henrik L. Frandsen, Ladan Parhamifar, Jannik B. Larsen, Thomas L. Andresen

**Affiliations:** ^1^ Center for Intestinal Absorption and Transport of Biopharmaceuticals, Technical University of Denmark, Lyngby, Denmark; ^2^ Department of Health Technology, Technical University of Denmark, Lyngby, Denmark; ^3^ The National Centre for Nano Fabrication and Characterization, DTU Nanolab, Technical University of Denmark, Lyngby, Denmark; ^4^ National Food Institute, Technical University of Denmark, Lyngby, Denmark

**Keywords:** organ-on-a-chip, drug transport, fluorescence live cell imaging, drug development, high through put screening platform

## Abstract

Unsuccessful clinical translation of orally delivered biological drugs remains a challenge in pharmaceutical development and has been linked to insufficient mechanistic understanding of intestinal drug transport. Live cell imaging could provide such mechanistic insights by directly tracking drug transport across intestinal barriers at subcellular resolution, however traditional intestinal *in vitro* models are not compatible with the necessary live cell imaging modalities. Here, we employed a novel microfluidic platform to develop an *in vitro* intestinal epithelial barrier compatible with advanced widefield- and confocal microscopy. We established a quantitative, multiplexed and high-temporal resolution imaging assay for investigating the cellular uptake and cross-barrier transport of biologics while simultaneously monitoring barrier integrity. As a proof-of-principle, we use the generic model to monitor the transport of co-administrated cell penetrating peptide (TAT) and insulin. We show that while TAT displayed a concentration dependent difference in its transport mechanism and efficiency, insulin displayed cellular internalization, but was restricted from transport across the barrier. This illustrates how such a sophisticated imaging based barrier model can facilitate mechanistic studies of drug transport across intestinal barriers and aid *in vivo* and clinical translation in drug development.

## 1 Introduction

Drug formulation development to allow for oral drug administration has been pursued for decades due to high patient compliance (Balimane et al., 2000; Masaoka et al., 2006). However, especially for biologics, such as peptides and proteins, the success has been limited and only five orally administered peptide formulations have reached the clinic (Drucker, 2020; Maher et al., 2021). These issues reflect the translational struggles seen broadly within the field of drug delivery, spurring the push for a deeper mechanistic understanding of drug transport, allowing for better rational design of drug formulations (Mechanism matters, 2010; Time to deliver, 2014).

To identify and understand the mode of action employed by drug formulations for crossing the intestinal barrier, assays compatible with live cell imaging techniques for monitoring the drug-cell barrier penetration in real-time would be ideal (Gumbleton, 2005; Watson, 2005; Larsen et al., 2021). The most popular and established *in vitro* platform for studying drug transport has been the Transwell system (TW), where cells are cultivated on a rigid membrane that separates two medium-containing chambers under static conditions (Hidalgo et al., 1989; Hubatsch et al., 2007). However, the structural build of TW limits its compatibility with live cell-imaging modalities (Zaderer et al., 2019), impeding high-resolution kinetic measurements and image-based readouts, and therefore provides only limited direct information on the underlying transport mechanisms. In addition, the TW system is incapable of incorporating three-dimensional cell cultures, extracellular matrix support and perfusion flow, all aspects shown to be highly important for cell proliferation and differentiation (Basson et al., 1996; Simon-Assmann et al., 2007; Huh et al., 2011; Youhanna and Lauschke, 2021). In recent years advanced microfluidic platforms were developed in order to recapitulate the three-dimensional physiology of the intestinal epithelial and the gastrointestinal environment *in vitro* (Bein et al., 2018). Among these, the OrganoPlate represents a user-friendly platform facilitating real-time fluorescent readouts. However until now, the OrganoPlate have primarily been used for toxicity assessment (Trietsch et al., 2017; Beaurivage et al., 2019), or disease modeling (Beaurivage et al., 2019). Only recently, did Hagiwara et al. apply the platform in the context of drug delivery as they investigated how the cross barrier transport of two well-known small molecule drugs was affected by simulated intestinal fluids. However, taking full advantage of the compatibility of the OrganoPlate with live cell imaging for studying the cross barrier transport mechanism of biologics is yet to be achieved. Furthermore, a prerequisite for this is a systematic validation of the OrganoPlate as a reliable *in vitro* model with a high *in vivo* drug absorption predictability by a direct correlation to standard drugs with known human absorption values. Expanding and validating models for use in transport studies is non-trivial, as highlighted by recent accounts showing that it is paramount to perform a comprehensive validation as they often are limited by poor abilities to predict the *in vivo* level of drug transport (Dahlgren et al., 2015). Together this highlights how there are still room for new methodologies facilitating the study of *in vitro* drug transport in validated, easy-to-use and reproducible platforms (Gleeson and McCartney, 2019), especially compatible with real-time fluorescent imaging (Wong et al., 2020; Halamoda-Kenzaoui et al., 2021; Larsen et al., 2021; Yilmaz et al., 2022).

Here, we employed the OrganoPlate microfluidic chip as a platform for developing an *in vitro* intestinal barrier model allowing us to study drug transport and uptake mechanisms using advanced fluorescent imaging modalities. An image-based in-chip validation of epithelial differentiation and polarization markers in generated monolayer cell tubules revealed a leak-tight, fully differentiated epithelial cell barrier, encompassing important features of the *in vivo* barrier, including a complex mucus layer and a functional metabolic machinery known to influence drug transport efficiency. We validate, for the first time, the microfluidic platform as a suitable model for intestinal drug transport studies by correlating the transport of small molecule drugs to the corresponding human absorption data. Furthermore, we use the model as a multiplexed live-cell imaging-based assay to obtain mechanistic in-depth knowledge about the mobility of biologics across fully differentiated and polarized cell barrier tubules. This epitomizes how the developed epithelial barrier system can facilitate sensitive real-time image-based quantitative studies on the transport and intracellular fate of biologics.

## 2 Materials and methods

### 2.1 Cell culture

The human colon adenocarcinoma cell line Caco-2 (European Collection of Authenticated Cell Cultures (0,9,042,001, ECACC) were maintained in Eagle’s Minimum Essential Medium (EMEM) (30–2003, ATCC) supplemented with 10% fetal bovine serum (FBS) (S1810-500, Biowest), 1% MEM non-essential amino acids (NEAA) (M7145, Sigma) and 1% penicillin/streptomycin (P/S) (P0781, Sigma). The clone HT29-MTX-E12 generated from the parental human colon adenocarcinoma cells (12,040,401, ECACC), HT29, were cultured in Dulbecco’s Modified Eagle’s Medium (DMEM) (D5796, Sigma) supplemented with 10% FBS, 1% NEAA and 1% P/S. Both cell lines were maintained at 37°C, 5% CO_2_ in a humidified incubator. Caco-2 cells with passage numbers between 47–61 and HT29-MTX-E12 cells between passage numbers 50–70 were used for all experiments. Cells were seeded for experiments after reaching 80–90% confluency in parental flasks. For coculture experiments, both cell lines were cultured in Caco-2 cell growth media.

### 2.2 OrganoPlate culture

For all experiments the three-lane OrganoPlate (4,004–400-B, Mimetas BV) with 40 individual chips was used, where each chip consists of a seeding channel, an ECM-channel and a perfusion channel (220 µm height x 300 µm width). Cell seeding into the OrganoPlate was performed as previously described (Trietsch et al., 2017). In brief, a 4 mg ml^−1^ Collagen 1 (3,447–020–01, R&D Systems) ECM-gel supplemented with 100 mM HEPES (H0887, Sigma) and 3.7 mg ml^−1^ NaHCO_3_ was prepared. The ECM-mixture was dispensed into the ECM-channel (2 µL) and placed in the incubator for 30 min to polymerize. After polymerization 30 µL Hanks’ Balanced Salt solution (HBSS) was added to the ECM-channel to avoid gel drying. Next, cells were detached with 0.25% trypsin EDTA and diluted to a final concentration of 1 × 10^7^ cells mL^−1^. For coculture of Caco-2 and HT29-MTX E12 cells, a ratio of 6:1 was used. The upper channel of the chip was filled with 2 μL cell suspension (a total of 20 × 10^3^ cells/channel) and the OrganoPlate was placed vertically inside the incubator for 3 h to allow cell attachment. Thereafter, 50 µL medium was added to the seeding channel (inlet and outlet) and the perfusion channel (inlet and outlet). The OrganoPlate was incubated horizontally for 4 days on an interval rocker (*±* 7° angle, 8 min rocking interval) at 37°C, 5% CO_2_ resulting in a formation of a tubule inside the chip. The medium was replaced every 2–3 days.

### 2.3 Transwell culture

Caco-2 cells were seeded at a final cell density of 5 × 10^4^ cells cm^−2^ in a 24-well format Transwell system with a surface area of 0.33 cm^2^ (CLS3413, Corning). The cells were grown in EMEM with 10% FBS and 1% P/S over 19–21 days and the medium was replaced every 2–3 days.

### 2.4 Transepithelial resistance measurements

The barrier integrity of cells seeded in the Transwell system was monitored routinely using an EVOM2 system equipped with an EndOhm-6G cup for measurements TEER (World Precision Instruments, Sarasota, FL). Raw resistance data were translated into TEER using the equation:

$$TEER\left[\Omega \times cm^{2}\right]=TEER\_RAW\left[\Omega \right]\times surface\;area\left[cm^{2}\right]$$ (1)

Caco-2 monolayers with a TEER above 1,500 Ω*cm^2^ were selected for further drug absorption studies.

### 2.5 Imaging modalities

Spinning disc (SD) confocal microscopy was performed on a Nikon Ti2 inverted microscope equipped with a Yokogawa CSU-W1 module and a Photometrics Prime 95B sCMOS camera was used for all experiments (excl. Image-based TAT transport). The set-up includes four excitation lasers 405/488/561/638 nm with 442/42, 520/28 BrightLine HC filters and 600/50 ET, 700/75 ET bandpass filters. For this work, a set of CFI Plan Apochromat Lambda objectives were used: ×4/0.2, 20x/0.75, 60x/1.4 and 100x/1.45. For image-based transport experiments, epi-fluorescence microscopy was performed using a Nikon Eclipse Ti2 microscope equipped with an LED source (CoolLED), a dual-band filter cube (AHF, Excitation 455–489 nm, 557–588 nm; Emission 506–542 nm, 605–660 nm) and a Photometrics Evolve 512 electron-multiplying charge-coupled device camera was used (10x N.A. 0.30).

### 2.6 Barrier integrity assay

The BI of the mono- and coculture systems was analyzed on day 1, 2 and 4. The Medium in the seeding channel was replaced with EMEM containing 0.5 mg ml^−1^ 4.4 kDa TD (T1037, Sigma) and placed on an interval rocker for 15 min in the incubator. After incubation, the plate was imaged using a SD confocal microscope at ×4 magnification. Inhomogeneous cell coverage in the seeding channel over during the cultivation leads to an inhomogeneous fluorescence intensity profile. To ensure an accurate comparison of BI values the whole channel (Seeding and ECM channel) was selected for measuring the intensity.

### 2.7 Immunostaining

Cells seeded in the OrganoPlate were fixed with 4% paraformaldehyde in phosphate-buffered saline (PBS) (D8537, Sigma) for 15 min, washed with PBS and permeabilized for 10 min in 0.3% Triton-X (T8787, Sigma). Subsequently, cells were washed with 4% FBS/PBS and incubated for 30 min in blocking solution 3% Bovine Serum Albumin (BSA)/PBS followed by incubation with primary antibody for 60 min at room temperature. Cells were then washed with 4% FBS/PBS and incubated for 30 min with the secondary antibody. Primary antibodies against Rabbit-a-Zonula occludens-1 (ZO-1) (61–7,300, Thermo Fischer, 1:200), Rabbit-a-Mucin 2 (Muc2) (PA5-21329, Thermo Fischer, 1:200), Mouse-a-Ezrin (610,602, BD Transduction, 1:100), Mouse-a-Multidrug Resistance Protein 2 (MRP2) (SC-59608, Santa Cruz, 100 µg ml-1), Mouse-a-Breast *Cancer* Resistance Protein (BCRP) (ab3380, Abcam, 1:50) were used. For the secondary antibody staining, Goat Anti-Rabbit IgG (H + L) Alexa Fluor 488 (ab150077, Abcam, 1:200) and Goat anti-Mouse IgG (H + L) Alexa Fluor 647 (A-21236, Thermo Fisher, 1:200) were used. Following antibody staining cells were washed three times with 4% FBS/PBS and nuclei were stained with 4′,6-diamidino-2-phenylindole (DAPI) (D1306, Thermo Fisher, 1:1,000) for 5 min. Cells were kept in PBS at 4°C and imaged using the previously described SD confocal microscope. All protein stainings were repeated at least three times in mono-and coculture tubules. Images in Figure 2 display representative immunostained cell-tubules.

### 2.8 Microvilli visualization using transmission electron microscopy

The coculture tubules were fixed by filling all three channels of the chip system with a solution of 2% glutaraldehyde, 4% paraformaldehyde in 0.1 M Na cacodylate buffer at pH 7.4. After fixation for 30 min at room temperature, the plate was stored at 4°C to await further processing. Each channel was washed three times with 0.1 M Na cacodylate buffer and stained with 1% osmium tetroxide in a 0.1 M Na-cacodylate buffer for 2 h at room temperature. After staining with osmium tetroxide, the channels were washed three times in milliQ water and stained with a 1% uranyl acetate solution overnight. The following day, the channels were washed twice in milliQ water followed by dehydration in increasing concentrations of ethanol, 50, 70, 95% and twice 100% with 20 min between solvent exchanges, to ensure complete dehydration. The channels were further dehydrated three times in acetonitrile for 10 min each before the start of resin infiltration. The channels were infiltrated with a 1:1 solution of TAAB 812 medium hardness embedding resin and acetonitrile. After 1 h, the channels were re-infiltrated with the same 1:1 solution to ensure complete infiltration. After one more hour, the channels were infiltrated with a 2:1 resin:acetonitrile solution overnight. The next morning the channels were re-infiltrated with the 2:1 solution for 6 h. The channels were then infiltrated with pure resin overnight. The following morning the channels were again re-infiltrated with pure resin to ensure complete infiltration. After 6 hours the plate was cured at 60°C for 72 h.

After the resin was completely cured, individual channel setups were cut from the larger plate for further processing. The samples were placed in a concentrated hydrofluoric acid solution, 48 wt%, to dissolve the glass substrate. After 45 min, the sample was removed from the hydrofluoric acid and rinsed with a saturated calcium chloride solution, 45 wt%. The sample was then rinsed with milliQ water. The now exposed channel was cut from the remaining device and re-embedded in TAAB resin which was cured overnight at 60°C. The resin block was trimmed with a razor blade, exposing a surface perpendicular to the channel. Thin sections, 120 nm thick, were cut using a Leica EM UC7 ultramicrotome and placed on a nickel slot grid coated with carbon-stabilized formvar. TEM was performed utilizing a FEI Tecnai T20 G2 TEM located at the Center for Electron Nanoscopy at the Technical University of Denmark, and images were acquired using TVIPS XF416 CCD camera.

### 2.9 Microvilli visualization using transmission electron microscopy

The coculture tubules were fixed by filling all three channels of the chip system with a solution of 2% glutaraldehyde, 4% paraformaldehyde in 0.1 M Na cacodylate buffer at pH 7.4. After fixation for 30 min at room temperature, the plate was stored at 4°C to await further processing. Each channel was washed three times with 0.1 M Na cacodylate buffer and stained with 1% osmium tetroxide in a 0.1 M Na cacodylate buffer for 2 h at room temperature. After staining with osmium tetroxide, the channels were washed three times in milliQ water and stained with a 1% uranyl acetate solution overnight. The following day, the channels were washed twice in milliQ water followed by dehydration in increasing concentrations of ethanol, 50, 70, 95% and twice 100% with 20 min between solvent exchanges, to ensure complete dehydration. The channels were further dehydrated three times in acetonitrile for 10 min each before the start of resin infiltration. The channels were infiltrated with a 1:1 solution of TAAB 812 medium hardness embedding resin and acetonitrile. After 1 h, the channels were re-infiltrated with the same 1:1 solution to ensure complete infiltration. After one more hour, the channels were infiltrated with a 2:1 resin:acetonitrile solution overnight. The next morning the channels were re-infiltrated with the 2:1 solution for 6 h. The channels were then infiltrated with pure resin overnight. The following morning the channels were again re-infiltrated with pure resin to ensure complete infiltration. After 6 hours the plate was cured at 60°C for 72 h.

After the resin was completely cured, individual channel setups were cut from the larger plate for further processing. The samples were placed in a concentrated hydrofluoric acid solution, 48 wt%, to dissolve the glass substrate. After 45 min, the sample was removed from the hydrofluoric acid and rinsed with a saturated calcium chloride solution, 45 wt%. The sample was then rinsed with milliQ water. The now exposed channel was cut from the remaining device and re-embedded in TAAB resin which was cured overnight at 60°C. The resin block was trimmed with a razor blade, exposing a surface perpendicular to the channel. Thin sections, 120 nm thick, were cut using a Leica EM UC7 ultramicrotome and placed on a nickel slot grid coated with carbon-stabilized formvar. TEM was performed utilizing a FEI Tecnai T20 G2 TEM located at the Center for Electron Nanoscopy at the Technical University of Denmark, and images were acquired using TVIPS XF416 CCD camera.

### 2.10 Mucus determination with alcian blue staining

Leak tight tubules in the OrganoPlate were fixed with 4% PFA for 15 min and washed afterwards three times with PBS. The cell tubules were acidified with 3% acidic acid and stained with Alcian blue (B8438, Sigma) for 30 min at room temperature. Next, cells were washed three times with PBS for 5 min and bright-field images were acquired with a light microscope.

### 2.11 P-glycoprotein mediated calcein efflux

Calcein-AM is a substrate of the P-gp efflux transporter. After internalization, it fluoresces until it is pumped out the cells (see Supplementary Figure S3). All channels in the OrganoPlate were washed with Opti-buffer containing Phenolred-free medium (DMEM) and HBSS (H6648, Sigma, Ratio of 1 DMEM: 3 HBSS). For detection of Calcein-AM efflux, the Opti-buffer in the seeding channel was replaced with Calcein- AM (C3099, 10 µM in 0.1% DMSO). Inhibition of the P-gp transporter was achieved by pre-incubation with Verapamil (V4629, Sigma, 50 µM in 0.5% Methanol) in the cell-seeding channel. The OrganoPlate was incubated for 60 min on an interval rocker at 37°C with 10 µM Calcein-AM or 50 µM Verapamil +10 µM Calcein-AM. To ensure that the presence of neither 0.5% methanol nor 0.1% DMSO affected the cross membrane partitioning of calcein-AM, both ± verapamil experiments contained the same amount of these compounds. Following incubation, all solutions in all inlets/outlets were replaced with ice cold stopping solution (20 µM Verapamil and nuclei stain Hoechst (H1399, Thermo Fischer, 10 μg ml^−1^). After 15 min of incubation the cells were imaged immediately by SD confocal microscopy using 405 nm (442/42 BrightLine HC emission filter) and 488 nm (520/28 BrightLine HC emission filter) excitation lasers using a ×20 air objective. Analyzing tubule intensity was performed in Fiji, and the whole tubule was selected as the region of interest (ROI) and the fluorescence intensities for both, calcein and nuclei were extracted. The ratio of calcein and nuclei fluorescence intensity was calculated, representing the intracellular fluorescence of the cell.

### 2.12 Aminopeptidase-N activity determination

Aminopeptidase-N activity of cells was determined using 1.5 μM l-Alanine 4-nitroanilide hydrochloride (A4N) (A9325, Sigma) as a substrate. Serum-free medium was used for the bottom flow channel and the medium in the seeding channel was replaced with A4N. The OrganoPlate was incubated on the interval rocker for 2 h at 37°C, 5% CO_2_. After incubation, the solution in the inlet and outlets of the seeding channels were collected and transferred to a 96-well plate. The absorbance of the cleaved product 4-nitroanaline was measured at 405 nm in a plate reader (Tecan, Switzerland).

### 2.13 *In vitro* drug absorption study

All drugs were purchased from Sigma Aldrich. Cells seeded in Transwells and OrganoPlate were washed with transport buffer (TB; 10 mM HANKS/HBSS). For the apical side, the pH of TB was adjusted to 6.5 (TB-apical) and for the basolateral side the pH was set to 7.4 (TB-basal). All drugs were diluted to a final concentration of 10 µM in 0.1% DMSO. The cultured monolayers were exposed to the drug for 2 h at 37°C, 5% CO_2_. Control cells were exposed to TB without drugs but supplemented with a final concentration of 0.1% DMSO on the apical side. The OrganoPlate was placed for the total 2 h of drug incubation under constant flow, whereas the Transwells were incubated for 2 h under static conditions according to standard protocols (Artursson and Karlsson, 1991; Pontier et al., 2001; Béduneau et al., 2014; Twarog et al., 2020). For the permeability quantification of each drug, samples were analyzed using LC-MS and correlated to a standard curve. As a control for barrier integrity, 100 µM Lucifer Yellow (LY) were added to all samples (Ayehunie et al., 2018).

### 2.14 Quantification of absorbed drugs with liquid chromatography mass spectrometry

Samples and standards were analyzed with a Shimadzu Nexera X2/Prominence HPLC (Shimadzu Europe, Duisburg, Germany) and ESI micrOTOF-Q III (Bruker Daltonics, Bremen, Germany) LC-MS setup. The LC was performed by injection of the analyte (5 µL) on a Poroshell 120 SB-C8 column, 2.7 µm, 2.1 × 50 mm (Agilent, Santa Clara, CA, United States) followed by elution with a linear gradient of MeCN and 2.5 mM NH_4_OH in water with 0.1% formic acid (from 0 to 100% over 9 min) at a flow rate of 0.4 ml min^−1^ (a detailed description of the HPLC setup and gradient is reported in Supplementary Table S1). The chromatographic front (1.75 min) was diverted to waste while the remaining run was injected into the ion source. A calibration solution consisting of 2.5 mM NaOH, 2.25 mM formic acid in 90% *i*-PrOH/water was injected into the ion source between 1.75 and 1.85 min at a flowrate of 30 μL h^−1^ for internal calibration of the spectra. MS analysis was performed in positive mode in the range 50–2,000 m *z* ^*−1*^ at a rate of 2 Hz. The MS settings used for analysis of the various drugs are listed in the Supplementary Table S2. The concentration determination of each individual drug was used to calculate the apparent permeability of a compound (apical to basolateral) according to the equation (Lennernäs et al., 1997):

$$P_{app}=\frac{dQ}{dt}*1/AC_{0}$$ (2)

Where dQ/dt is the flux of drug across the cell monolayer per time [µM sec^−1^], A is the surface area of the monolayer exposed to the drug (cm^2^) and C_0_ is the initial concentration of the drug.

A two-tailed non-parametric Spearman’s correlation function was applied to determine the Spearman correlation coefficient R (von Erlach et al., 2020). The R-value defines the correlation between the quantified *P*_*app*_ of the drugs in the used model with the known human absorption values, a value closer to one represents a stronger correlation. Furthermore, a four-parametric logistic model curve fit was applied (Sjöberg et al., 2013) to better visualize the correlation between the quantified *P*_*app*_ values and the known human absorption.

### 2.15 Image-based localization and quantification of TAT and TAT/INS transport across epithelial monolayer tubules

Coculture tubules were used on day 4 after seeding and after confirming their leak-tight BI. Varying concentrations of TAT (24 μM; 2 µM) were applied to the coculture-tubules for capturing the internalization. Here, the cells were counterstained with Hoechst (H1399, Thermo Fischer, 10 μg ml^−1^) and incubated for 1 h at 37°C, 5% CO_2_, washed with PBS and images were acquired using SD confocal microscopy with 488 nm laser and 520/28 BrightLine HC filters for detecting FITC-labelled TAT and the 405 nm laser and 442/42 BrightLine HC filter for acquiring Hoechst. The cell tubules exposed to TAT/INS (24 µM/50 μM, IS1-AF647-1, Nanocs) were stained with above mentioned cellular dyes and CellMask-green (C37608, Thermo Fisher, 1:500) for 30 min on ice after 3 h of incubation. The transport of TAT was tracked over 2 h, acquiring time lapse images every 30 s using epi-fluorescence microscopy (see Imaging modalities). Two different concentrations of TAT (24 μM; 2 µM) or TAT/INS were applied to the coculture chips and imaged. To simultaneously monitor the barrier integrity TD (0.5 mg ml^−1^) was co-applied to the chips and measured in parallel to TAT. For the quantification of both TAT, INS and TD transport across the cell barrier, calibration curves were established. The rate of transport was extracted by a linear fit of each individual curve (Supplementary Figures S7, S8). In-house developed MATLAB based macro was used for analysis.

### 2.16 Image based analysis of salcaprozate sodium (SNAC) induced semaglutide transport

Coculture tubules were exposed to SNAC (20 mM or 40 mM) in combination with semaglutide-Cy3 (5 µM) and FITC-dextran (46,944, Sigma Aldrich, 0.5 mg ml^−1^) and imaged over approx. 4 h as described above.

## 3 Results

### 3.1 Development and verification of leak-tight small intestinal chip model

**FIGURE 1:**
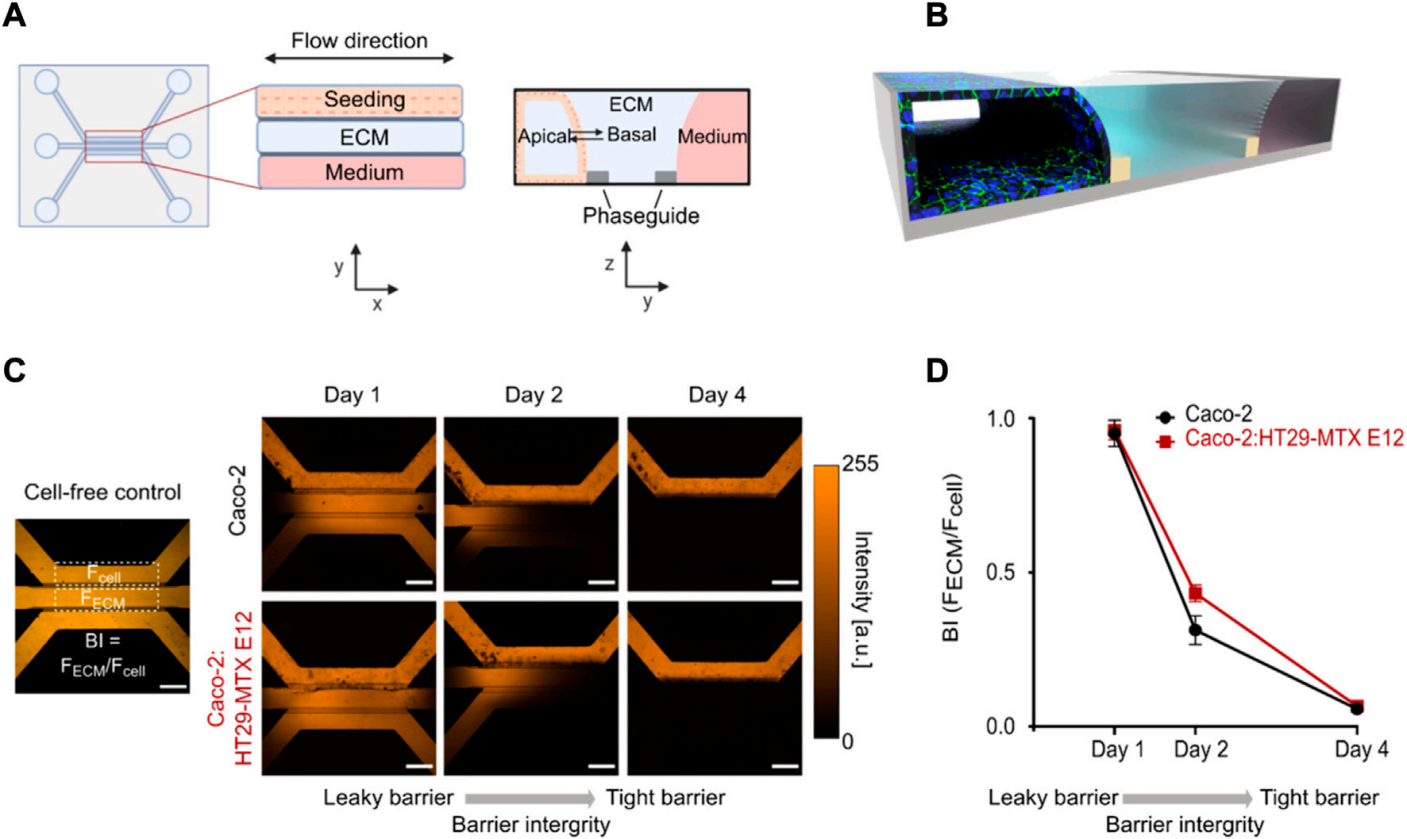
Mono- and coculture systems produce leak-tight epithelial monolayer tubules after 4 days. **(A)** Design of the 3-lane OrganoPlate system with a top view (left) and side view (right) illustrated in 2d. **(B)** 3D-schematic of the one chip after the formation of an epithelial monolayer tubule stained for tight junctions (green) and cell nuclei (blue) in the seeding channel. **(C)** Micrographs displaying TD distribution in cell free control (left) and in perfused mono-and coculture chips at days 1, 2, 4 post seeding (right). **(D)** Quantifying the BI of mono- and coculture tubules by depicting the BI determined as the ratio between the TD intensity in the ECM channel (F_ECM_) and seeding channel (F_cell_) (see C) as a function of days post cell seeding. Error bars represent the standard deviation (*n* = 3). Scale bar in all micrographs is 500 µm.

To develop an imaging compatible intestinal *in vitro* model we utilized the OrganoPlate, constituting a 348-Microwell set up with 40 individual chips and a 150 µm thick glass bottom (Trietsch et al., 2017; Gijzen et al., 2020). The chips are constructed with a seeding channel, an extracellular matrix (ECM) channel and a (fluidic) perfusion channel (Figures 1A,B). To enable the use of the chip as a model for studying intestinal transport, we first ensured that an intact and tight cellular barrier was formed. We seeded either pure Caco-2 cells (Monoculture) or a 6:1 ratio of Caco-2:HT29-MTX E12 cells (Coculture), the latter in an attempt to increase the biological relevance through the presence of mucus-producing goblet cells. For both systems, we facilitated the formation of a differentiated epithelial monolayer by inducing constant shear stress of 0.13 Pa and a mean flow rate of 2.02 μL min^−1^ using a pump free perfusion system for the total culturing period of only 4 days (Schutgens et al., 2019). To evaluate the barrier integrity (BI) we then applied a 0.5 mg ml^−1^ solution of 4.4 kDa Tetramethylrhodamine isothiocyanate (TRITC)-dextran (TD) in the seeding channel and used confocal microscopy to image the TD distribution in the chip. If no cells were added to the chip the TD intensity spread through the whole chip, a distribution also seen for both mono- and coculture systems after 1 day of barrier formation (Figure 1C). After 2 days we recorded a partial decrease in the TD intensity in the ECM and medium channels, while after 4 days both mono- and coculture chips displayed complete restriction of TD intensity to the seeding channel, demonstrating that a tight barrier had formed (Figure 1C, Day 4). In comparison, culturing of HT29-MTX E12 cells as a monoculture did not result in the formation of a tubules structure capable of restricting TD diffusion (Supplementary Figure S1). To quantitatively verify the presence of a tight barrier after 4 days of incubation, we determined the BI value by extracting the integrated intensity inside a region of interest in the ECM- and seeding channels (Figure 1C). We calculated the BI value for each chip as F_ECM_/F_cell_. This allowed us to ascertain cell barrier tightness, with values ranging from 1 (leaky) - 0 (tight), noting that BI values below 0.4 have previously been defined to represent a tight barrier (Trietsch et al., 2017) (Figure 1D). On day one post cell seeding we quantified BI_Mono_ = 0.95 ± 0.07 and BI_Co_ = 0.96 ± 0.05, constituting leaky cell barriers. After 2 days in culture BI_Mono_ = 0.32 ± 0.08 and BI_Co_ = 0.43 ± 0.05 values were calculated, representing a semi-tight cell barrier (Figure 1D, Day 2). On day four post seeding, the low TD intensity in the receiving channels lead to BI_Mono_ = 0.05 ± 0.01 and BI_Co_ = 0.07 ± 0.01 values, verifying that for both systems a tight barrier was established (Figure 1D, Day 4).

### 3.2 The epithelial tubule represents a fully polarized and differentiated cell monolayer

**FIGURE 2:**
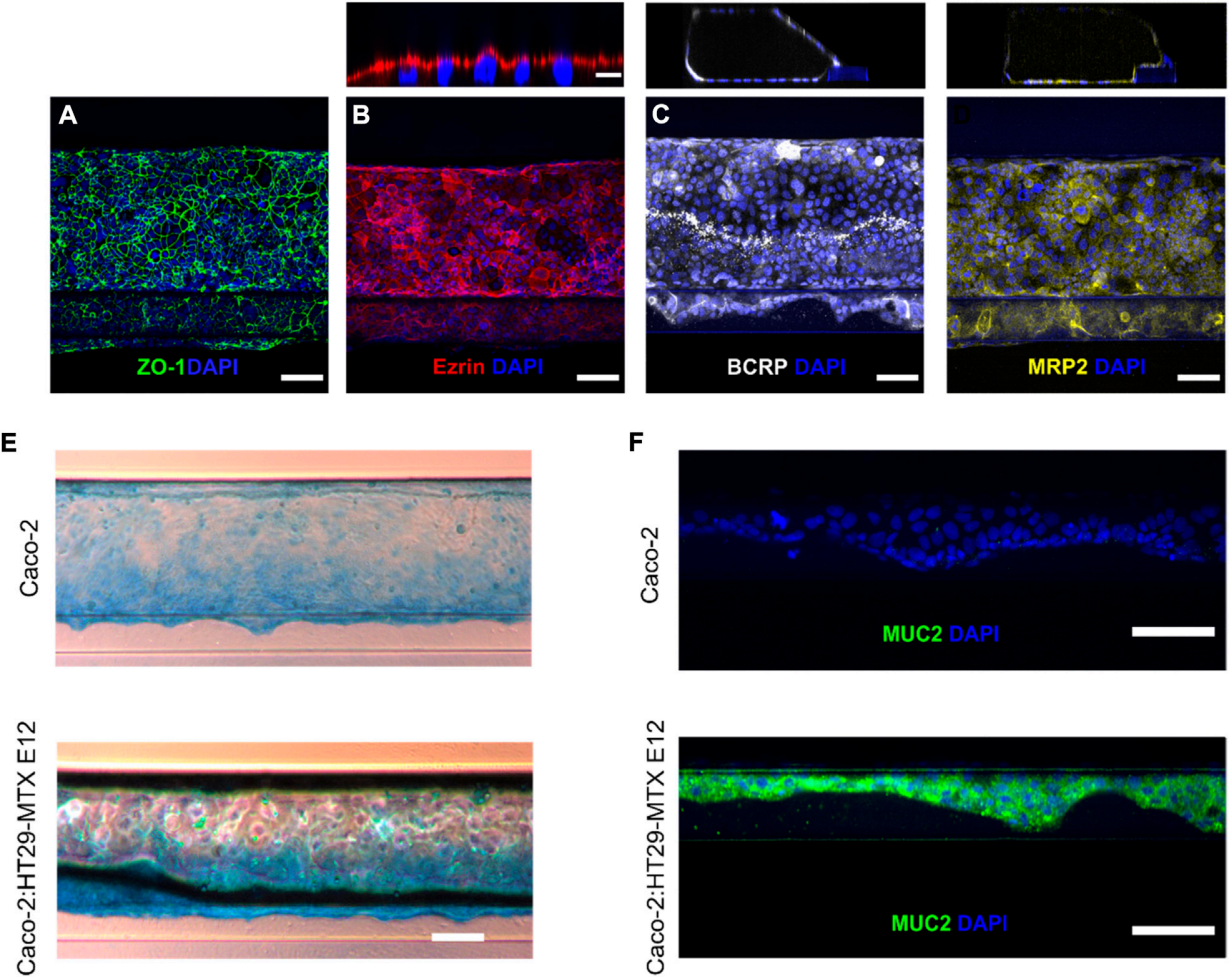
Verification of a differentiated and polarized epithelial monolayer using direct in-chip immunostaining. **(A)** Tight junction formation depicted by maximum intensity projection (MIP) from confocal z-stack of ZO-1 (green) and DAPI (blue) (scale bar is 100 µm). **(B)** Bottom, brush borders are visualized as MIP from confocal z-stack of ezrin (red) and DAPI (blue) (scale bar is 100 µm). Top, the apical orientation of ezrin expression is depicted as a vertical cross-section of the cell-tubule (Scale bar is 10 µm). **(C)** Bottom, BCRP efflux transporter (white) and DAPI (blue) are visualized as MIP from confocal z-stack (scale bar is 100 µm). Top, A vertical cross section of the BCRP stained cell-tubule. **(D)** Bottom, MIP of cell-tubule stained for the efflux transporter MRP2 (yellow) (scale bar is 100 µm). Top, vertical cross section of the MRP2 stained cell-tubule. **(E)** Brightfield microscopy images showing Alcian Blue staining of acidic mucins in mono- (top) and coculture (bottom) tubules (scale bar is 200 µm). **(F)** Micrograph of single z-slice of the cell layer along the ECM interface for mono- and coculture depicting Muc2 (green) and DAPI (blue) (scale bar is 100 µm).

To confirm the polarization and differentiation of the cell-tubules into an epithelial monolayer we relied on conventional staining strategies for characteristic phenotypical epithelial cell markers (Anderson et al., 1989; Sambuy et al., 2005; Englund et al., 2006). The unique ability of the microfluidic platform of immunostaining and imaging directly in the system bypasses the need for cell monolayer extraction necessary in traditional *in vitro* TW models. Both culture systems revealed a homogenous expression of the tight junction protein Zonula occludens-1 (ZO-1) along the whole tubule, demonstrating its differentiation into a compact epithelial cellular network (Figure 2A). We next determined the apicobasal membrane orientation of the cell-tubules by immunostaining the known apically expressed brush border protein ezrin (Cao et al., 2014). Performing z-stack imaging allowed us to investigate ezrin staining in vertical cross-section of the cell tubule (Figure 2B, top). For all individual cells, ezrin staining was completely restricted to the cellular membrane facing the tubule lumen, as determined by a distinct fluorescence signal of ezrin above the cell nucleus (Figure 2B). This demonstrates that the cellular tubules are differentiated into monolayers of correctly polarized epithelial cells. Next, we identified two major intestinal efflux transporters of the ABC-family, the Breast *Cancer* Resistance Protein (BCRP) and the Multi Drug Resistant Protein (MRP2), which are key regulators of drug localization and thus essential for the *in vitro* platform accurately predicting intestinal transport (Giacomini et al., 2010; Billat et al., 2017). By staining for BCRP and MRP2 we observed the expected expression profile along the whole monolayer tubules, strengthening the conclusion that the cells are fully differentiated (Figures 2C,D). All apicobasal membrane orientation assessments described above were performed at least three times in both mono- and coculture tubules finding equivalent expression and localization of ZO-1, ezrin, MRP2 or BCRP for both systems (Supplementary Figure S2). Overall, the in-chip immunostaining revealed the formation of a fully differentiated and polarized epithelial tubule.

### 3.3 Homogenous Muc2-expression in coculture-tubules along the extracellular matrix-cell interface

To evaluate if introducing the goblet cells in the coculture induced the expected mucus production we performed various imaging-based assays to evaluate the mucus expression in both the mono- and coculture system. First, we studied the presence and expression profile of unspecific mucins by staining the cultured cell-tubules with Alcian blue, which binds to all acidic mucins (Figure 2E, Supplementary Figure S2) (Béduneau et al., 2014). The coculture tubules showed a notable higher mucus expression compared to the monoculture tubule (Gijzen et al., 2020). This was especially observed along the ECM-interface, highlighting the need for an ECM support to promote adequate cell differentiation. To verify the presence of Muc2, which is the small intestinal specific mucus type produced by goblet cells (Schneider et al., 2018), we visualized Muc2 by confocal imaging of immunostained cell-tubules. The staining for Muc2 proteins showed a strong and homogenous expression in coculture tubules but was completely absent in monoculture tubules (Figure 2F). Again, it was primarily the cells supported by the ECM that displayed high expression of Muc2 in coculture tubules. Thus, the introduction of the goblet-like cells in coculture systems leads to strong and homogeneous mucus production in contrast to Caco-2 cells in monoculture. This addition increases the biological relevance of the epithelial monolayer system (Karlsson et al., 1993; Hilgendorf et al., 2000; Doherty and Charman, 2002) and permits its use for studying how the mucus layer affects drug transport across the intestinal cell barrier, by comparing the mono and coculture tubules.

### 3.4 Microvilli formation in coculture tubules corroborates an *in vivo* mimicking cellular morphology

**FIGURE 3:**
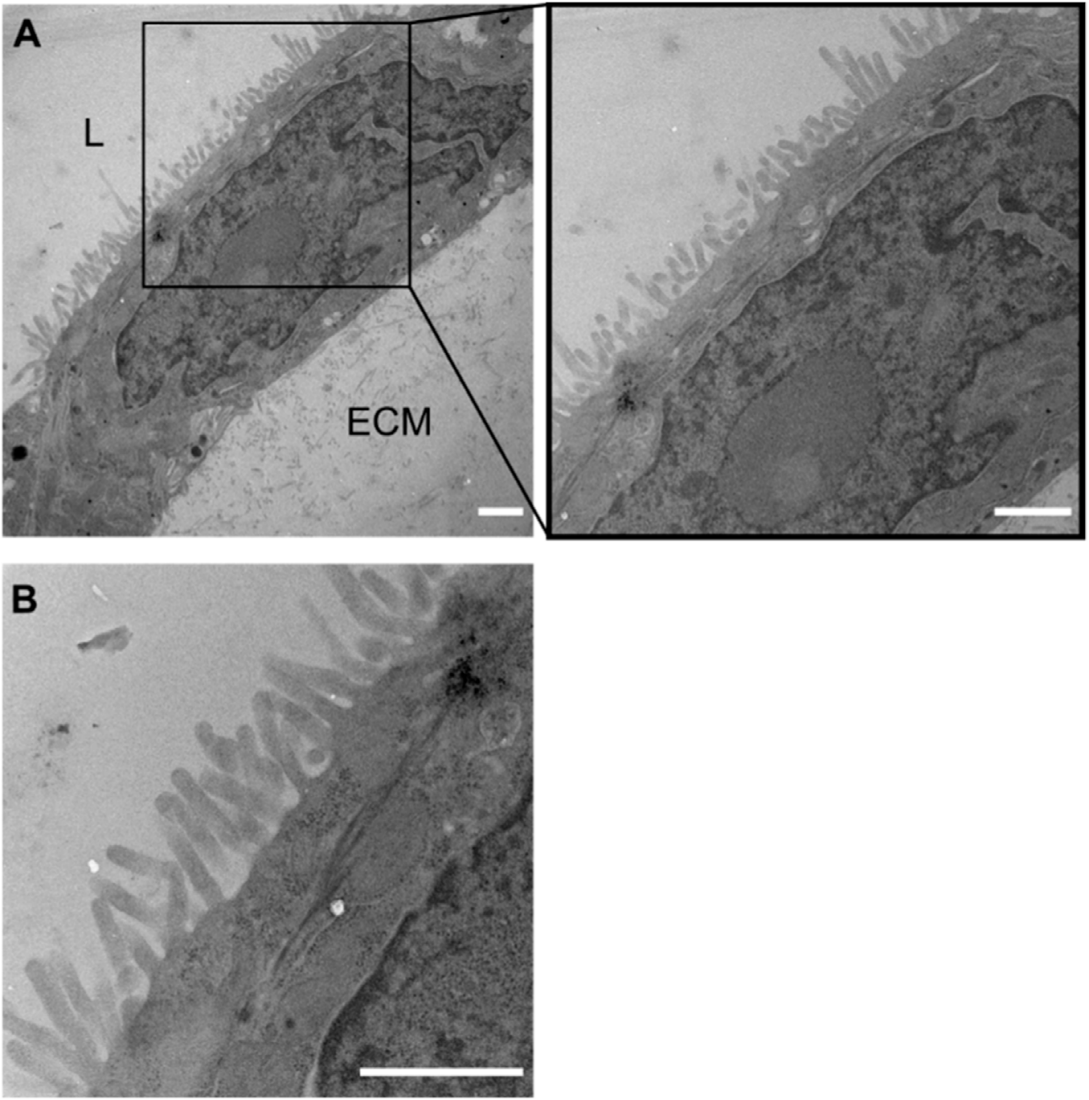
Formation of microvilli in coculture tubules identified by TEM. **(A)** Micrograph of a monolayer cell tubule with the lumen top left (marked by L) and the ECM located towards the bottom right (marked by ECM). Clear microvilli formation towards the luminal side of the coculture tubule, but a complete lack of microvilli on the side facing the ECM demonstrating the correct apicobasal morphological differentiation after 4 days of culture. **(B)** Zoom **(A)** represents the organization of dense microvilli. Scale bar is 1 µm in all images.

To further corroborate the correct polarization of the cell monolayer we visualized cell morphology in detail by extracting the coculture tubule samples and imaging them using transmission electron microscopy (TEM) (Figure 3). Cell tubules were fixed, stained, embedded and sectioned into 120 nm thick slices after 4 days of culture before the tubule sections of the cell-ECM interface were imaged. The micrographs of the tubule sections displayed a distinct and homogenous formation of microvilli along the whole tubule, restricted only on the side facing the lumen opposite to the ECM, clearly illustrating the development of a well-defined apical side (Figure 3A). The basal side of the cells, which is attached to the ECM did not show any morphological changes. Additionally, each individual cell expressed a dense network of microvilli and thus a membrane surface area expansion typically seen for differentiated intestinal epithelial (Figure 3B). Having this characteristic cellular morphology in an *in vitro* assay is pivotal for its *in vivo* absorption predictability as the cell membrane surface area is a strong regulator of the total drug absorption. Thus, the generated cell tubules fully differentiated into epithelial cells evidenced by their apicobasal characteristic membrane morphologies.

### 3.5 Intestinal metabolic markers are expressed in mono- and coculture tubules

**FIGURE 4:**
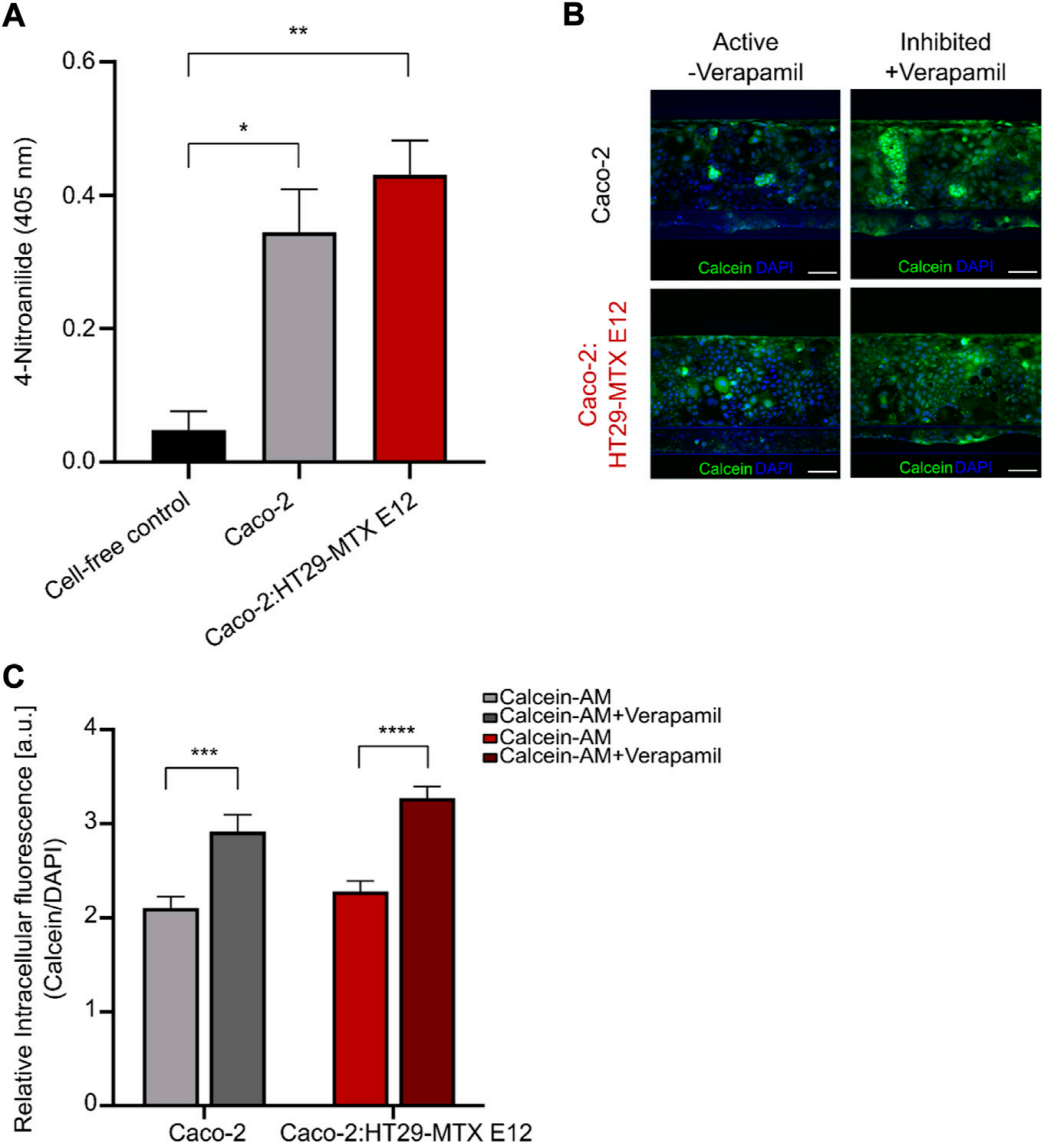
Identification of active intestinal metabolic enzyme and P-gp transporter in mono-and coculture tubules. **(A)** Brush border enzyme aminopeptidase N quantification in cell free (black), monoculture (grey) or coculture (red) systems measured as the absorbance of the cleavage product 4-Nitroanailide. Error bars represent the standard error of the mean (SEM) of at least three biological replicates per condition (*p < 0.05, **p < 0.005). **(B)** Left, micrographs depicting the non-inhibited calcein (green) and DAPI (blue) fluorescence intensities after 1 h incubation for monoculture (top) and coculture (bottom). Right, micrographs depicting the Verapamil-inhibited calcein (green) and DAPI (blue) fluorescence intensity after 1 h incubation for monoculture (top) and coculture (bottom). Scale bar is 100 µm. **(C)** Quantification of the relative intracellular fluorescence (I_relative)_ in non-inhibited monoculture (light grey), Verapamil-inhibited monoculture (dark grey), non-inhibited coculture (light red) and Verapamil-inhibited coculture (dark red). Error bars represent the SEM of at least three biological replicates per condition. (*p = 0.047, **p = 0.0019, ***p = 0.0004, ****p < 0.0001).

To establish a physiological relevant small intestinal *in vitro* platform for drug transport studies the presence and activity of proteolytic enzymes and drug transporters are crucial (Gan and Thakker, 1997; Langguth et al., 1997; Estudante et al., 2013). A highly relevant group of intestinal metabolic enzymes are the aminopeptidases, therefore we evaluated the presence of the brush border aminopeptidase N by employing a standard absorbance based assay, measuring the enzymatic cleavage product 4-Nitroanilide (Figure 4A) (Kim et al., 2012). Both mono- and coculture tubules showed a significant increase in measured 4-Nitroanilide compared to the cell-free control, confirming the presence of functional aminopeptidase N in both mono- and coculture tubules after 4 days of culture.

After having verified the presence of intestinal efflux transporters (Figures 2C,D) we wanted to ensure that these also constituted a functional efflux transport machinery, therefore we examined the function of the intestinal P-glycoprotein (P-gp) efflux transporter by following the change in intracellular calcein upon inhibition with Verapamil (Figures 4B,C, Supplementary Figure S3). We added 10 μM of calcein to the tubular lumen and incubated for 60 min before imaging the whole tubule (Figure 4B, active). The relative intracellular fluorescence (I_relative_) was then determined as the ratio of the calcein and DAPI intracellular fluorescence intensities for the whole tubule. For both the mono- and coculture systems we found similar low I_relative_ values for mono- and coculture tubules, due to the continuous efflux of calcein mediated by the active P-gp transporter (Figure 4C, light grey (mono) and light red (coculture)). Next, we repeated the experiment while adding the P-gp inhibitor Verapamil leading to a significant increase in I_relative_ values for both the mono- and coculture systems (Figure 4B, Inhibited; Figure 4C, dark grey (mono) and dark red (coculture)). This demonstrated the presence of functional P-gp transporters, representing a key component of the dynamic efflux machinery known to affect drug transport and thus vital for creating an *in vitro* model with high *in vivo* transport predictability.

### 3.6 Mono and coculture tubules display strong *in vivo* drug transport predictability

**FIGURE 5:**
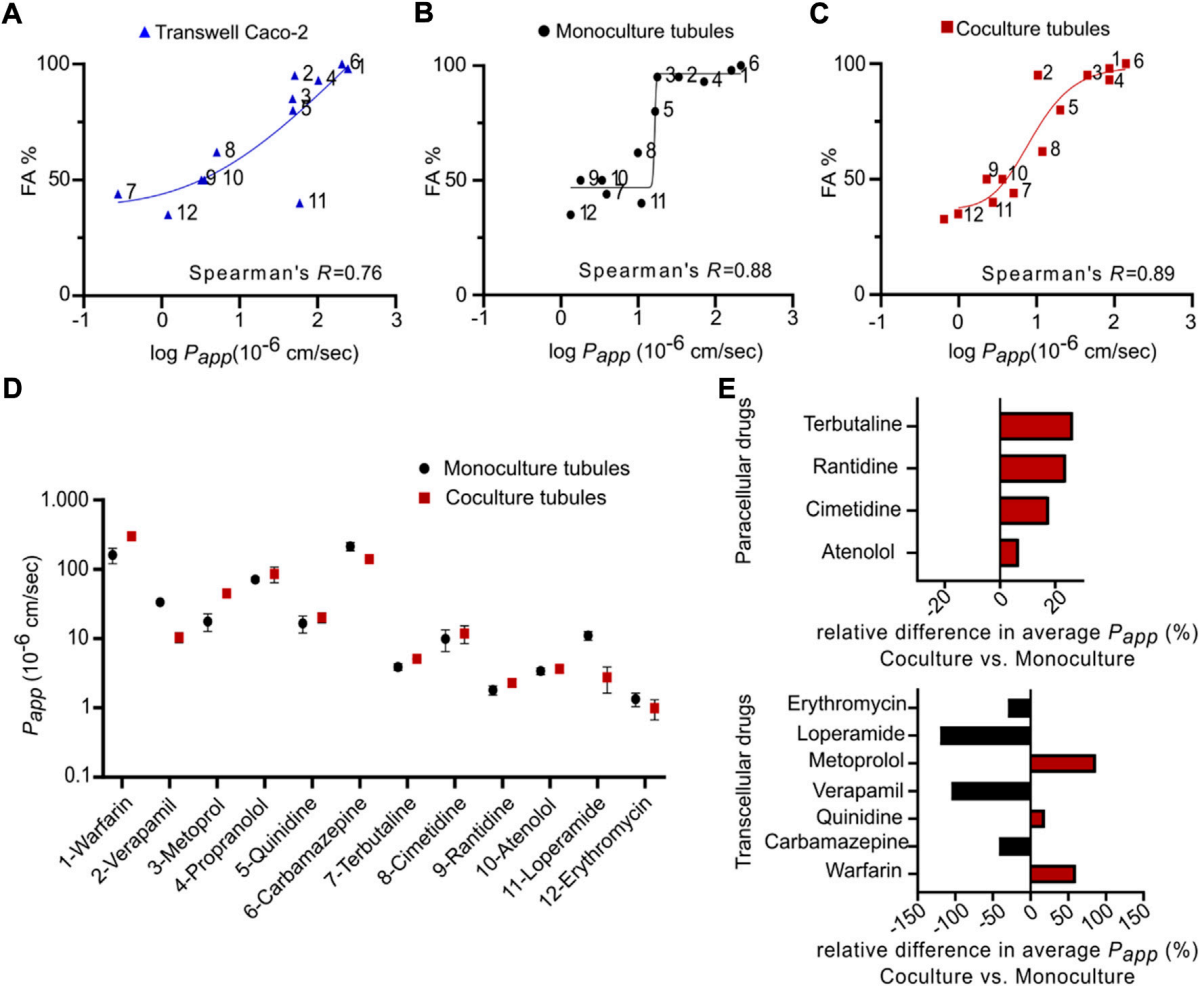
Correlation between fractions absorbed in human and apparent permeability coefficient of twelve model drugs in mono- and coculture tubules as well as standard Transwells. **(A–C)** The FA for each drug plotted against the average log *Papp* obtained from **(A)** Transwell (blue) **(B)** monoculture tubules (black) and **(C)** coculture tubules (red). All fits represent non-linear regression analysis using a two-tailed non-parametric Spearman’s correlation function, generating Spearman’s coefficients (*R*). Compounds 1–12 are listed in Supplementary Table S4. **(D)** The average *P*_*app*_ found for either the monoculture (black) or the coculture (red) tubules plotted for each individual drug tested. **(E)** The relative difference of the average *P*_*app*_ for paracellular transported drugs (top) and transcellular transported drugs (bottom). Error bars represent the SEM for each drug transport measured for at least three biological replicates (*n* = 3).

To validate the chip platform for drug transport studies, we correlated the absorption of twelve small model drugs in both the mono- and coculture tubules to their known human absorption values. To ensure a complete evaluation across the whole range of known human absorption values, as recommended by the Food and Drug Administration (FDA), we selected model drugs from all Biopharmaceutical Classification System (BCS) classes with quantifiable transport (Supplementary Table S4) (Amidon et al., 1995; Niazi, 2019). For comparison, we performed the same correlative analysis for a classical TW system (Lipka and Amidon, 1999), which was set up following standard protocols (see experimental section). To quantify the transport, we added the drugs to either chips or TW and extracted both the apical and basolateral fractions after 2 h (Supplementary Figure S4, Supplementary Tables S3, S4). The concentration in all samples was determined using liquid chromatography mass spectrometry (LC-MS) and internal standards (see experimental section, Supplementary Tables S1, S2). The apparent permeability (*P*_*app*_) of each drug was calculated as described in the method section and summarized in Supplementary Table S4. Comparing the TW and the monoculture tubules systems we quantified ranges of *P*_*app*_ values from 1.20 × 10^−6^ cm s^−1^ for Erythromycin to 243.41 × 10^−6^ cm s^−1^ for Warfarin in TW and 1.33 × 10^−6^ cm s^−1^ for Erythromycin to 213.99 × 10^−6^ cm s^−1^ for Carbamazepine in the chip monoculture. We correlated the obtained permeability data of each system to the known human absorption values (Cheng et al., 2007; Skolnik et al., 2010; Takenaka et al., 2016; Youhanna and Lauschke, 2021) by plotting the *P*_*app*_ values against the fraction absorbed in humans (FA) and extracted the Spearman correlation coefficient (SCC-R) (von Erlach et al., 2020). This statistic represents a measure of how well an *in vitro* model recapitulates the *in vivo* transport and thus allows for comparison between models (Figures 5A,B) (Li et al., 2007; Skolnik et al., 2010; Takenaka et al., 2016). We determined a SCC-R of 0.77 for the TW system, indicating a strong correlation to *in vivo transport*. In addition, the transport in monoculture tubules showed a similar SCC-R to TW with 0.88. This demonstrates that the monoculture is a qualified platform for drug transport studies, with an ability to predict *in vivo* intestinal transport at least on the level of the widely employed TW system, additionally having the unique ability to be directly compatible with advanced imaging modalities.

It has previously been shown that the utilization of Caco-2 monocultures for drug transport studies suffers from 1) underestimation of transport through the paracellular route due to a high density of tight junctions and 2) an overestimation of passively absorbed drugs due to the lack of mucus (Hilgendorf et al., 2000; Pontier et al., 2001; Béduneau et al., 2014). This underscores the importance of developing more biological complex *in vitro* models like the coculture tubules described in this work. Like for the TW and monoculture tubules, we next measured the transport of the twelve drugs in the coculture platform using LC-MS and quantified the *P*_*app*_ (Supplementary Table S4). The detected ranges of *P*_*app*_ values for the coculture system were from 0.99 × 10^−6^ cm s^−1^ for Erythromycin to 300.39 × 10^−6^ cm s^−1^ for Warfarin. Plotting the FA against the *P*_*app*_ for the coculture revealed a SCC-R of 0.89, which is overall similar to the values found for the monoculture tubules and TW (Figure 5C). Thus, while the coculture system did not directly improve the *in vivo* predictability as compared to the TW system it is encouraging that this more physiologically relevant model can be established in a platform compatible with advanced imaging. Also, to further compare the mono- and coculture tubule system and delineate the effect of introducing the goblet cells we performed a direct comparison of *P*_*app*_ (monoculture) versus *P*_*app*_ (coculture) for each individual drug (Figure 5D). No systematic trend towards elevated or diminished transport for one system in particular, was evident (Figure 5D), however, knowing the transport route employed by the individual drugs allowed us to selectively plot the relative difference in average *P*_*app*_, using a Bland-Altman comparison analysis (Altman and Bland, 1983). For all four paracellular transported drugs we saw increased relative transport for the coculture (red) versus the monoculture (black), up to 27% seen for Terbutaline (Figure 5E, top). This corroborates the believed increase in the monolayer permeability introduced when adding another cell line into the Caco-2 monolayer, directly increasing paracellular drug transport (Hilgendorf et al., 2000). Plotting the same relative difference in average *P*_*app*_ for the transcellular drugs, we did not see any systematic change towards elevated or diminished transport for either the mono- (black) or the coculture (red) tubules (Figure 5E, bottom, Supplementary Table S5). This suggests that the presence of a mucus layer only had a minute effect on the transcellular transport. However, we caution on underestimating the importance of the mucus layer based on experiments with only small molecule drugs. We imagine that for larger peptide and protein drugs, the presence of a mucus layer might strongly affect the transport rate and thus give a more biological accurate measure of their transport across an intestinal cell layer. Based on this, we choose to continue with the coculture tubule for subsequent transport studies of biologics.

### 3.7 Multiplexed live cell imaging technique on coculture tubules allows for simulations assessment of cellular uptake, transport and barrier integrity

**FIGURE 6:**
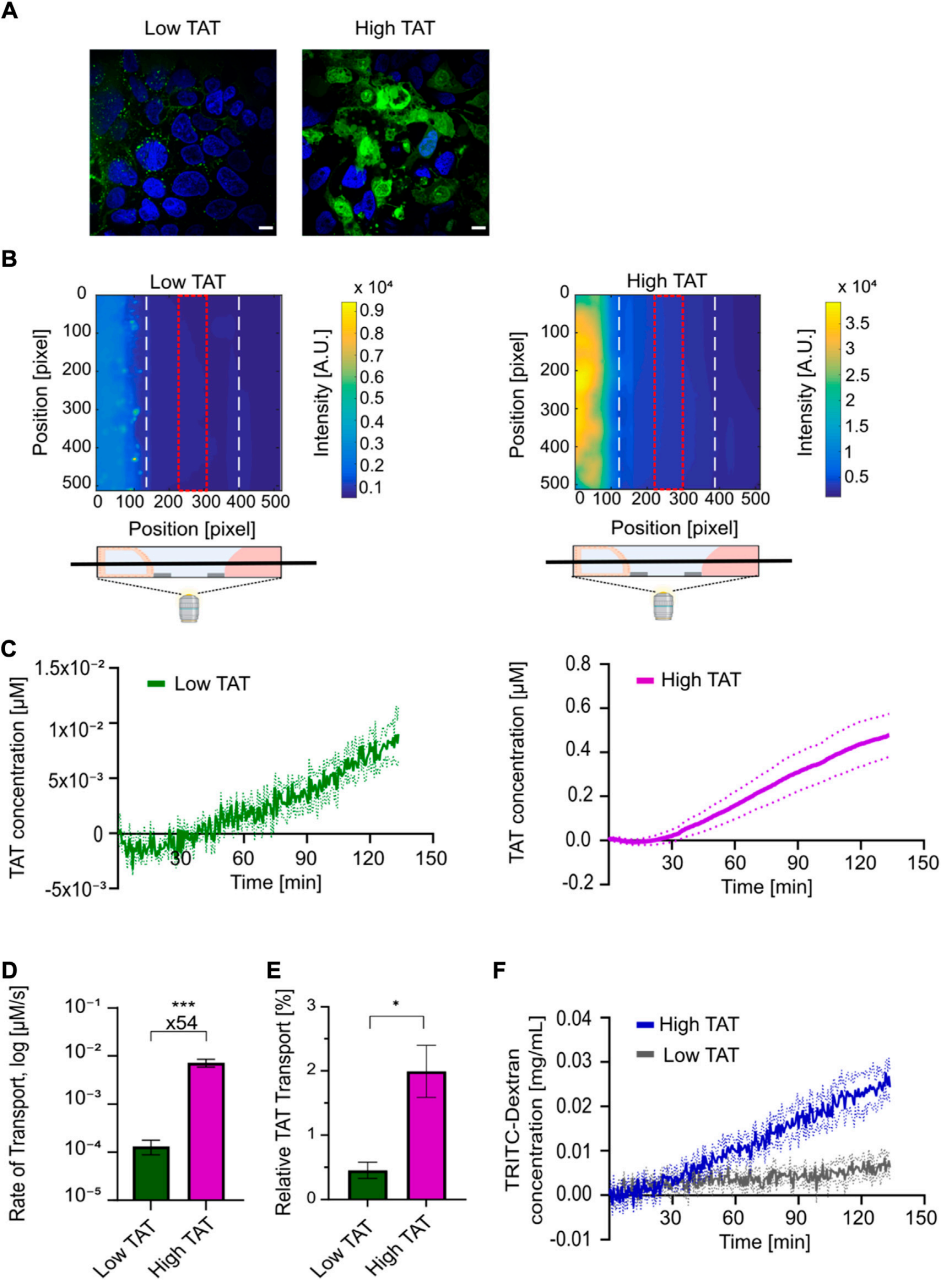
Live cell imaging-based uptake and transport quantification of TAT for determining high-temporal resolution kinetic transport profiles reveals a strong concentration dependent TAT transport in coculture tubules. **(A)** Live cell micrographs depicting TAT (green) and Hoechst (blue) intensities in coculture tubules treated with (left) low concentration of TAT (2 µM) or (right) high concentrations of TAT (24 µM). Scale bar is 10 µm. **(B)** Representative fluorescence surface intensity plot after 130 min incubation with the low TAT (left) and high TAT (right) concentrations. The surface intensity plots are taken from time series acquired using high-temporal resolution live cell imaging of TAT transport. The field of view was set as a z-plane in the middle of each chip-system, including all three connected channels as depicted. The fluorescence intensity summed along the red dotted line at the center of the ECM-channel was selected for quantification of transport. **(C)** Image-based quantification of kinetic transport profile plotted as the average TAT concentration within the red square in **(B)** as a function of time for both the low (green) and high concentration (purple) of TAT (*n* = 4). Dashed lines represent the SEM of each condition. **(D)** Comparison of the average rate of TAT transport for low and high TAT concentration systems. (*n* = 7) (***p = 0.0002). **(E)** Comparison of the relative TAT transport after 130 min of incubation (*n* = 4) (*p = 0.01). **(F)** Determination of BI kinetic quantified using the TD concentration for the low (grey) and high (blue) TAT concentration systems (*n* = 4). Dashed lines in the figure represent the SEM. All error bars in the figure represent the SEM of each condition.

We next used the developed and verified coculture tubules to use imaging-based live cell assays to untangle the uptake and transport mechanism of biologics. To validate the platform for quantitative image based intestinal transport studies, we first employed the FITC-labeled transcriptional activator peptide in HIV (TAT), an arginine-rich cell-penetrating peptide (CPP) extensively used to deliver therapeutic proteins or peptides across cellular barriers (Morris et al., 2001; Kristensen and Nielsen, 2016; Guo et al., 2019). While the ability of TAT to cross cellular-membranes and barriers are uniformly reported, the transport mechanism of TAT remains debated, potentially due to previously studies being restricted to fixed cell samples or undifferentiated single cell experiments (Frankel and Pabo, 1988; Brock, 2014). Here, the unique imaging capabilities of the cell-tubule platform allowed us to quantify, for the first time, TAT transport across a differentiated cellular barrier using high-temporal live cell imaging. We first investigated how the TAT concentration affected its transport mechanism by applying either a low (2 µM) or a high (24 µM) concentration of TAT to the cell-tubules and imaged the coculture chips after 1 h of incubation. We detected a clear concentration dependent difference in the intracellular localization of TAT, with the low TAT concentration system displaying a punctate distribution of TAT inside the cells, suggesting endosomal uptake (Figure 6A). In contrast, the high TAT concentration (24 µM) system displayed a diffuse TAT intensity in the cytoplasm, indicating uptake predominantly via transduction (cell membrane accumulation and destabilization) into the cells (Figure 6A). These results corroborate previous studies on undifferentiated single cell models, showing a concentration dependence of the cellular internalization mechanisms of TAT (Tünnemann et al., 2006, 2008; Duchardt et al., 2007; Brock, 2014). Here, we expand this to a fully differentiated coculture system demonstrating that concentrations differences down to a factor of 12 can lead to distinctly different uptake mechanisms of TAT.

Next we used the platform to study the barrier transport kinetics of TAT. We performed whole-chip real-time live cell imaging, to quantify the concentration of TAT transported across the coculture cell layer and reaching the receiving channel (Figure 6B). Here, we took advantage of the high temporal resolution and strong detection sensitivity offered by the imaging-compatible chip system. The same two concentrations of TAT (2 μM, 24 µM) were added to coculture tubules along with a 0.5 mg ml^−1^ concentration of TD, allowing us to simultaneously track barrier integrity over time. Both the FITC and TRITC channels were recorded for each tubular system every 30 s for a total of 2.5 h. The well-plate format of the microfluidic platform allowed us to mark the position of numerous coculture tubules and use automated multi-spot imaging to perform several experiments in parallel. A calibration experiment where known concentrations of TAT or TD were added to a chip without cells allowed us to convert the measured intensities to actual concentration in μM and mg mL^−1^ respectively (Supplementary Figure S5). A TAT surface intensity plot of the seeding, ECM and perfusion channels after 130 min of incubation showed a clear intensity gradient in the ECM-channel for both concentrations, demonstrating that TAT is capable of crossing the tubular cell layer and migrate through the ECM (Figure 6B). To quantify the transport of TAT we integrated the intensity within the ECM facing the receiving channel in the region displayed in Figure 6B (red square). We then used the calibration control to convert this intensity to concentration, which we plotted as a function of time for both the low- and high TAT concentration systems (Figure 6C). For both systems, we record a lag time of approx. 30 min showing a high noise to signal ratio, indicating the detection limit of the individual molecules before a significant signal above the background was detected. Subsequently, both systems reach a linear regime displaying a steady increase in transported peptide. Thus, to decouple the transport efficiency from the initial difference in concentration we quantified the transport rate for the linear regime for both the low and high TAT systems (Supplementary Figures S6, S7). Calculating the average transport rate for all experiments revealed a 54-fold higher rate for the high versus the low TAT system (Figure 6D). To elucidate how this affected the overall amount of transported TAT we again normalized for the initial difference in concentration by converting the quantified TAT concentration after 2.5 h to a percentage of the initially added concentration of TAT. Doing this, we quantified average relative transport percentages of 0.5 ± 0.1% for low TAT and 2.0 ± 0.4% for high TAT, demonstrating a 4.4 fold higher relative amount of TAT transported in the high versus low TAT concentration system (Figure 6E). Finally, the influence of TAT on the barrier integrity was displayed using the acquired TD time series, showing an intact barrier indicated by an overall transport of TD below 5%. However, the sensitivity of the assay allows to detect a TAT concentration dependent difference in the TD transport profile. In the high TAT system a steady increase of TD was observed, but only a minimal transport of TD was detected in the low TAT system (Figure 6F). This TAT concentration dependent differences in the barrier interaction, further support that TAT can use two very different transport pathways, governed by the initial TAT concentration. Additionally, our data demonstrate that the transduction pathway employed by TAT at higher concentrations is vastly more efficient than the endocytic pathway employed at lower concentrations.

### 3.8 Intracellular entrapment of insulin restricts its transport across the epithelial cell barrier

**FIGURE 7:**
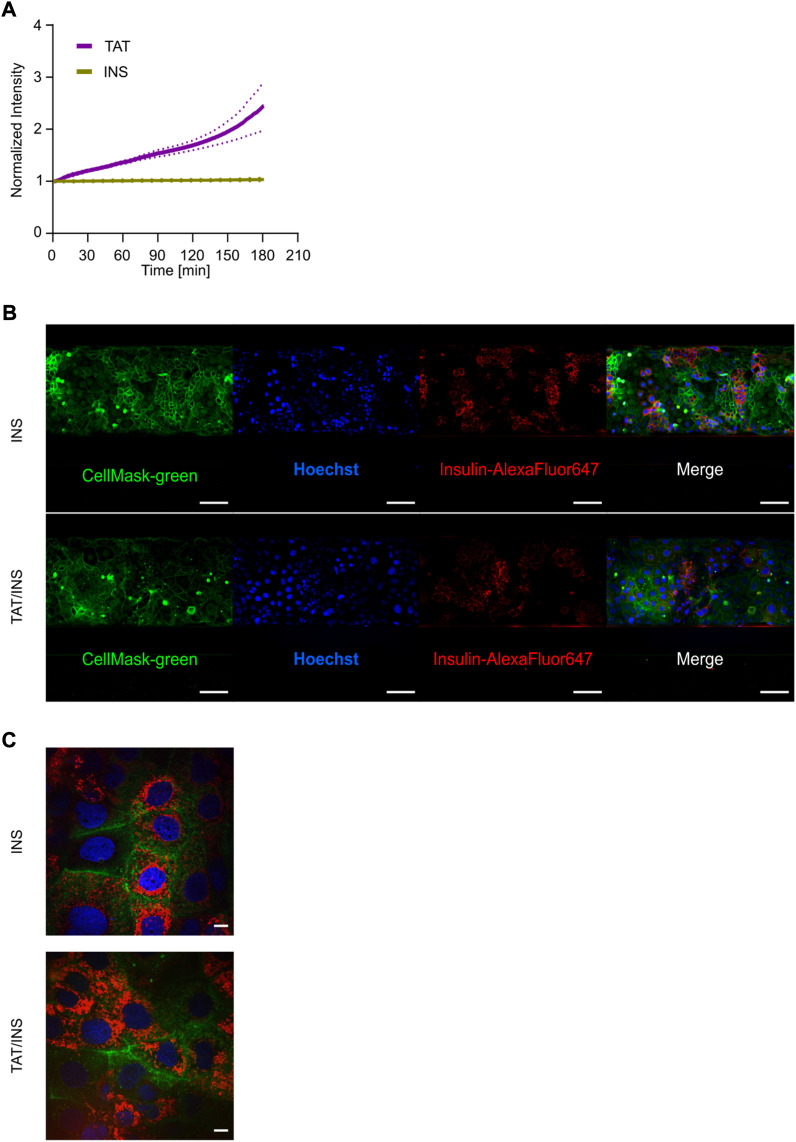
Multiplexed live cell imaging for assessing the intracellular localization and quantitative transport of Insulin. **(A)** Normalized intensities of INS (yellow) and TAT (purple) transport over 3 h. Data reflect the average of three independent biological replicates depicted with the SEM in dashed lines (*n* = 3). **(B)** Live cell micrographs showing the intracellular distribution of INS (red) with (bottom) and without (top) co-incubation of TAT for 3 h in coculture tubules. Tubules were stained with CellMask-green (green) for representation of the plasma membrane and Hoechst (blue) as nuclei staining. Scale bar is 500 µm. **(C)** The uptake and intracellular localization of INS with (left) and without the addition of TAT after 3 h of incubation. Scale bar is 10 µm.

Insulin is considered to be at the forefront of oral drug delivery, but despite immense efforts, no insulin-based oral formulation has reached the market, primarily due to extremely low bioavailability, originating both from low stability of free insulin in the harsh gastro-intestinal environment and an extremely low intrinsically cross barrier transport efficiency (Goldberg and Gomez-Orellana, 2003). Next, we took advantage of the subcellular resolution of the platform to study 1) the transport of AlexaFluor647-labeled insulin (INS) alone or 2) the transport of INS when using TAT as a vehicle for the attempted delivery across the cell barrier. In the latter case, we relied on the well-established strategy of electrostatic CPP and peptide complexation (Chen et al., 2017; Guo et al., 2019) and thus simultaneously added both TAT and INS to the tubule system. Additionally we added TD allowing us to track, in real-time, the transport of both the drug delivery vehicle (TAT) and the cargo (INS), while also monitoring how these affected the barrier integrity (TD). We detected no INS transport across the cell barrier independent of whether or not TAT was present, whereas a clear transport of TAT was again detected (Figure 7A). Also here, the barrier integrity was not compromised significantly in chips with and without TAT (Supplementary Figure S8). To ascertain the lack of INS transport we investigated its fate along the transport pathway after 3 h. We described the distribution of internalized INS by live cell imaging of the tubules showing that INS was indeed internalized into the enterocyte cell layer even without TAT (Figure 7B). Additionally, to investigate the intracellular we changed the magnification from ×20 to 100x for live cell imaging, allowing us to elucidate that for both INS and INS/TAT, the INS was localized inside the cytosol in punctual patterns, indicating endosomal uptake (Figure 7C). This suggests that the main limiting factor for the INS delivery across the cellular barrier in our setup was an inability to escape endosomes and/or be moved across the basolateral membrane.

Additionally, we attempted to use the model to study other pharmacological relevant transport mixtures including the permeation enhancer SNAC and the glucagon-like peptide 1 analog semaglutide (SG) used in the recently FDA approved treatment for type II diabetes (Buckley et al., 2018). However, from tracking the transport of SG-Cy3 and the BI by TD, in combination with a direct visual inspection of the cell layer in the bright field channel, we concluded that before any significant semaglutide-Cy3 transport across the barrier could be detected, SNAC had caused irreversible damage to the cell layer (Supplementary Figure S9). This illustrate the substantial impact on barrier morphology and integrity imposed by SNAC and suggests that its *in vivo* feasibility strongly relies on the regenerative nature of the epithelium cell layer. Overall, these findings illustrate the immense potential of employing imaging modality compatible *in vitro* intestinal barrier models, offering the ability to simultaneously perform detailed studies on uptake mechanisms and high-quality quantitative kinetic transport measurements and with that obtaining valuable mechanistic insights.

## 4 Discussion

Drug screening platforms compatible with live-cell imaging could offer the possibility to directly track how biologics cross the intestinal epithelial barrier and hereby offer mechanistic insights facilitating the rational design of new and improved oral administrated drugs (Gumbleton, 2005; Watson, 2005; Mechanism matters, 2010; Time to deliver, 2014; Sahay et al., 2010; Larsen et al., 2021). Here we developed a fully polarized and differentiated *in vitro* model from mono- and coculture epithelial tubules in the microfluidic OrganoPlate system. Both systems exhibited the correct expression patterns of standard epithelial cell markers such as brush border enzymes and efflux-transporters after 4 days of culture. In comparison, Caco-2 cells grown in Transwells require 17–21 days of culture for the differentiation into epithelial cells, proposed to be due to their static culture condition, as the importance of shear stress on Caco-2 cells for distinct and fast differentiation into an epithelial monolayer was reported in previous studies (Shemesh et al., 2015; Langerak et al., 2020). In our model, we applied bidirectional flow during the whole cultivation time and the resulting shear stress on the cell tubules induced the fast differentiation kinetics, greatly reducing the period from initial cell seeding to having a functional model for experiments.

In coculture tubules, we visualized and quantified the real-time transport of peptide transport using fluorescent microscopy. We employed TAT, a well-known vehicle for cellular drug delivery (Kristensen and Nielsen, 2016; Zhu et al., 2016), due to its proven ability to translocate across cell membranes, resulting in a push for identifying its underlying mechanism (Wadia et al., 2004; Brooks et al., 2005). Initially, it was proposed that TAT was mainly transported to the nuclei, but this was later disputed as concerns about potential artefacts from sample fixation were raised (Richard et al., 2003). Therefore, to avoid fixation investigators turned to live imaging of single cells, however, such systems do not accurately represent a biological cell barrier and are neither differentiated nor polarized cells. Therefore, we here investigated the uptake of TAT in the fully differentiated and polarized cell layer with mucus and microvilli, revealing a concentration dependent mode of uptake, corroborating earlier findings (Brock, 2014). Then we expanded this by showing that the difference in uptake mechanism also led to a significant concentration dependent difference in transport efficiency, quantifying a 54-fold increase in transport efficiency for a 12-fold difference in initial TAT concentration. This illustrates how our system can circumvent many of the shortcomings of previous *in vitro* models and provide detailed imaging based insights on the cellular uptake and barrier transport of peptides.

The finding that co-delivery of TAT/insulin does not lead to strong transport of insulin is in line with previous studies (Kamei et al., 2008), whereas the features of the platform enabled to disclose the intracellular accumulation and entrapment of insulin in endosomal compartments, despite TATs ability of performing endosomal escape mechanisms and hence being transported across the epithelium (Lönn et al., 2016). This demonstrates the versatility of the advanced model allowing for simultaneous mechanistic studies on uptake and intracellular transport as well as sensitive kinetic measurements of barrier translocation.

While the microscopy-based readout offers great advantages for tracking the transport of biologics, it also poses a limitation to the current setup, as the selected drug needs to be modified with a fluorescent reporter. It is known that the modification could potentially alter the transport behavior of biologics, especially for smaller peptides (Szeto et al., 2005; Hedegaard et al., 2018). Consequently, it is paramount to select fluorescent dyes that to a minimal extent interfere with the physicochemical property of the biologics and e.g. select one of the number of fluorophores that has been shown to display minimal propensity for interaction with lipid membranes (Hughes et al., 2014). Additionally, while the applied Caco-2 cell line is a workhorse within the *in vitro* intestinal barrier community, it is still not capable of fully replicating the complex cellular environment seen *in vivo* epithelium (Englund et al., 2006; Maubon et al., 2007; Harwood et al., 2016; Vaessen et al., 2017). Thus, the biological complexity of the model could potentially be increased by replacing the commonly used Caco-2 and HT29 cell lines with a more biological relevant cell pool, e.g. from intestinal organoids (Bein et al., 2018; Beaurivage et al., 2020; Naumovska et al., 2020; Pimenta et al., 2022).”

Due to the intrinsic low bioavailability of most biologics, the transport of orally delivered formulations across the intestinal cell layer typically requires some extent of barrier disruption (Brayden et al., 2020). Therefore, most oral biologics formulations include excipients that can increase the transport across the cellular barrier (Brown et al., 2020). To facilitate safe transport using this strategy for oral drug delivery it is crucial that the barrier disruption is only transient and can be fully reversed to avoid inducing long term degradation of the intestinal tissue (Maher et al., 2021). Therefore, monitoring barrier integrity is a cornerstone of traditional Transwell assay, however high-temporal measurements of the transient cell barrier disruption are difficult in Transwell setups. Consequently, specialized transepithelial resistance (TEER) instruments with the ability to record the barrier integrity with a temporal resolution down to seconds have to be employed (Srinivasan et al., 2015; Gerasimenko et al., 2020). However, these setups have major drawbacks including being expensive and have limited experimental throughput as simultaneous monitoring of transport and TEER are unfeasible. The real-time read-out feature of the tubule platform presented here allowed for continuous evaluation of the barrier integrity by tracking the TD intensity with high temporal resolution. Additionally, we demonstrated that such BI measurements can run simultaneously with drug transport studies without the measurements affecting each other, as long as the chosen fluorophores do not display significant spectral overlap. This possibility of multiplexing within the same experiment greatly increases the throughput and flexibility of the assay.

In summary, the developed cell tubule setup represents a novel *in vitro* model system of the small intestine epithelium with a very high *in vivo* predictability and is thus directly applicable for drug transport studies. The unique compatibility of the platform with common microscopy modalities for live cell imaging allowed for studying the uptake mechanism and real-time transport of TAT and insulin across a fully differentiated epithelial barrier with high sensitivity and temporal kinetics. The generic ability of the model to perform detailed mechanistic studies for all fluorescently labeled biologics underscores how its widespread implementation could greatly benefit early-stage oral drug development.

## Data Availability

The original contributions presented in the study are included in the article/Supplementary Material, further inquiries can be directed to the corresponding authors.
