# Supplementary material for: Quantifying the transport of biologics across intestinal barrier models in real-time by fluorescent imaging
Source: Front Bioeng Biotechnol. 2022 Sep 9;10:965200. doi: 10.3389/fbioe.2022.965200 (PMC9500407; doi:10.3389/fbioe.2022.965200)
Supplement: Supplementary file 1 [file DataSheet1.pdf]

Supplementary Material for manuscript:

Quantifying the transport of biologics across intestinal barrier models in real-time by fluorescent imaging

Arjen Weller, Morten B. Hansen, Rodolphe Marie, Adam C. Hundahl, Casper Hempel, Paul J. Kempen, Henrik L. Frandsen, Ladan Parhamifar, Jannik B. Larsen * *and Thomas L. Andresen**

Supplementary Figures:

1. Barrier Integrity determination for tubules prepared solely from HT29-MTX E12 cells reveal they do not produce a leak-tight barrier
2. Immunostaining of epithelial differentiation markers in monoculture tubules and alcian blue staining of mucin protein in mono- and coculture tubules
3. Illustration of image-based determination Pgp-efflux transporter by inhibition with Verapamil
4. Intact barrier function after 2 h of drug exposure on Transwells.
5. Concentration curves of TAT and TRITC-Dextran for image-based quantification of TAT transport
6. Linear regime extracted from the kinetic profile of individual high concentration TAT transport over time
7. Linear regime extracted from the kinetic profile of individual low concentration TAT transport over time
8. Intact barrier integrity after TAT/INS exposure assed by simultaneous FITC-dextran acquisition
9. Barrier integrity impairment as the rate limiting step for SNAC induced semaglutide transport

Supplementary Tables:

1. HPLC gradient
2. Conditions for drug quantification by LC-MS
3. Barrier function determination after drug exposure using Lucifer Yellow (LY) in chips with mono- and coculture tubules and Transwells
4. Comparison of apparent permeability in chips using mono-and coculture tubules and Transwell system of 12 model drugs
5. Direct statistical comparison of drug transport in Transwells mono-and coculture tubules of 12 model drugs using an unpaired t-test.

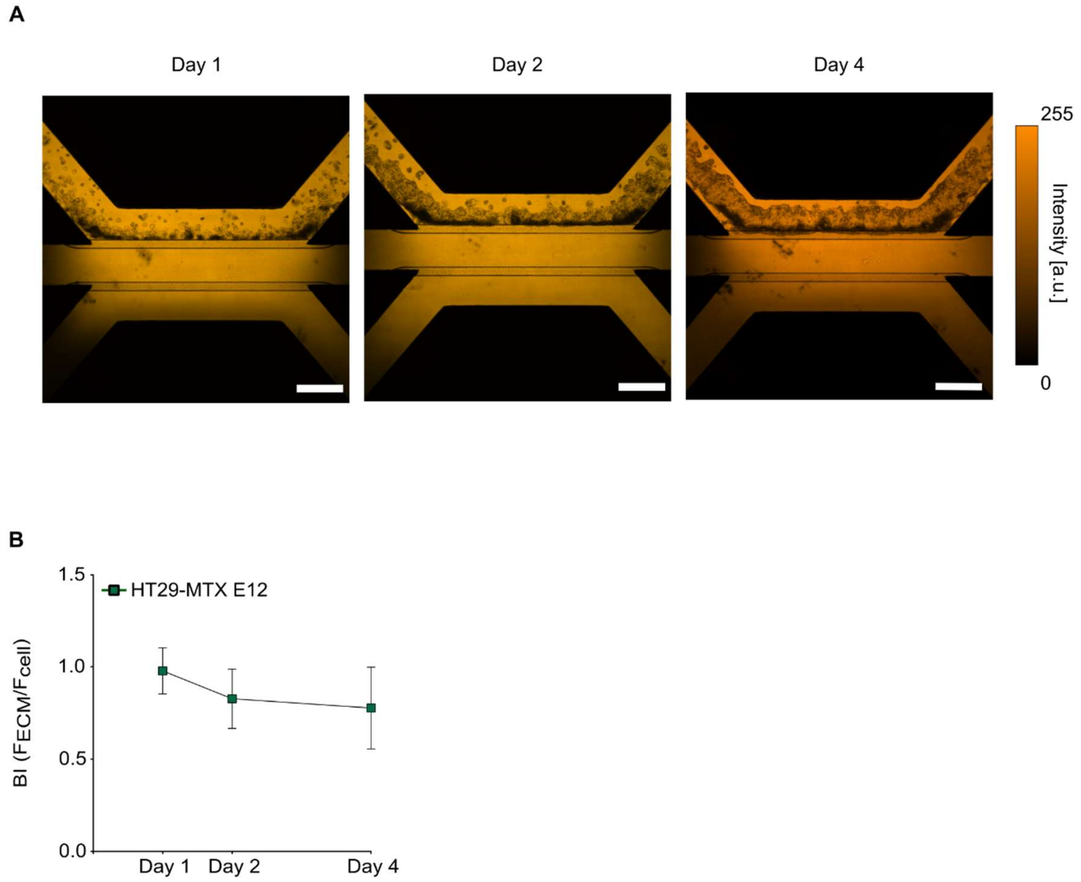

Supplementary Figure 1. **Barrier Integrity determination for tubules prepared solely from HT29-MTX E12 cells reveal they do not produce a leak-tight barrier.** (**A**) Micrographs displaying TD distribution in HT29-MTX E12 chips at day 1, 2, 4 post seeding. (**B**) Quantifying the BI of HT29-MTX E12 chips by depicting the BI determined as the ratio between the TD intensity in the ECM channel (F_{ECM}) and seeding channel (F_{cell}) as a function of days post cell seeding (see Figure 1c for equation). No barrier formation was detected over 4 days of culture. The Error bars represent the standard error of the mean (n=3). Scale bar in all micrographs are 500 µm.

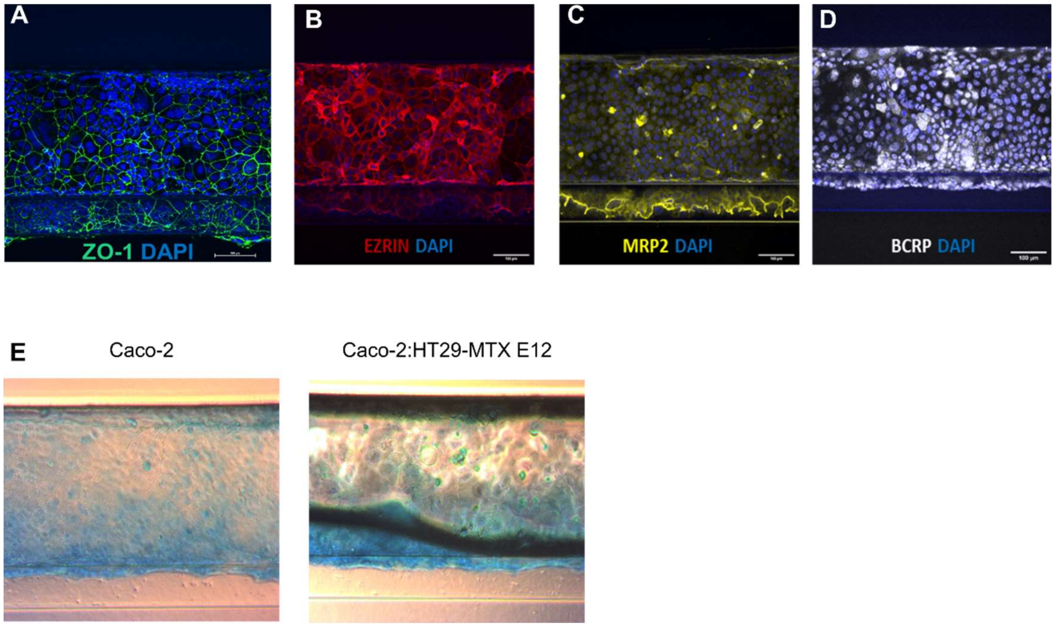

Supplementary Figure 2. Immunostaining of epithelial differentiation markers in monoculture tubules and alcian blue staining of mucin protein in mono- and coculture tubules. (**A**) TJ formation depicted by MIP from confocal z-stack of ZO-1 (green) and DAPI (blue) (scale bar is 100 μm). (**B**) Brush borders visualized as MIP from confocal z-stack of ezrin (red) and DAPI (blue) (scale bar is 100 μm). (**C**) MIP of cell-tubule stained for the efflux transporter MRP2 (yellow) (scale bar is 100 μm). (**D**) BCRP efflux transporter (white) and DAPI (blue) visualized as MIP from confocal z-stack (scale bar is 100 μm). (**E**) Brightfield microscopy images showing alcian blue staining of acidic mucin proteins in mono- (top) and coculture (bottom) tubules (20x magnification). All tubules were fixed on day 4 of culture.

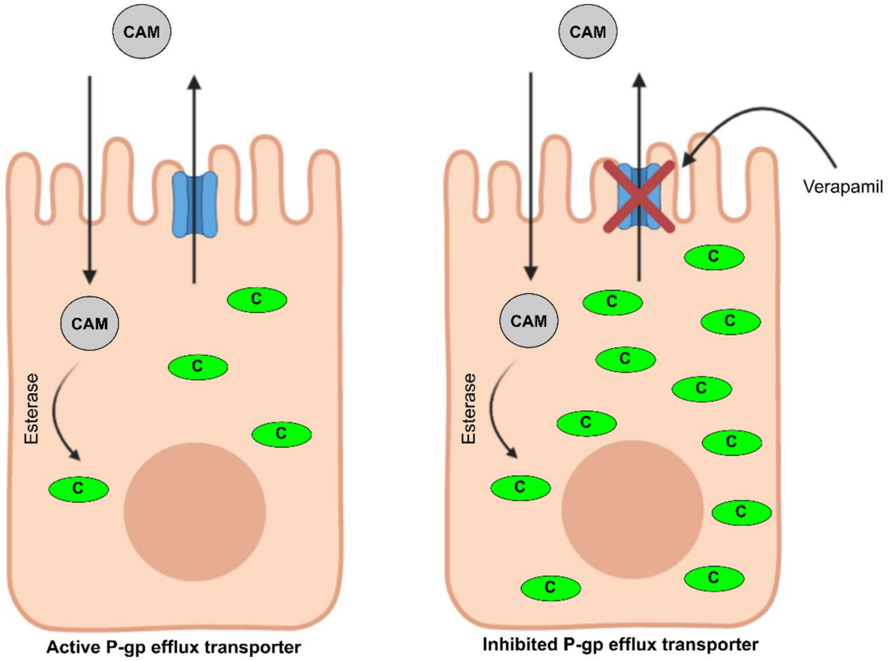

Supplementary Figure 3. Illustration of assay for determining P-gp efflux transporter activity through selective inhibition with Verapamil. To determine the presence and activity of the P-gp efflux transporter calcein-AM (CAM) was added to the cell tubules. Calcein-AM can diffuse into cells and subsequently be hydrolyzed to calcein (C), which results in an intracellular fluorescence signal. However, CAM is a substrate of the P-gp efflux transporter, and thus will be continuously pumped out of the cell in the presence of a functional P-gp machinery before the conversion into C. The equilibrated influx and efflux of CAM can be inhibited by Verapamil. The inhibition of the P-gp transporter leads to an increase in the intracellular C signal, which can be monitored by fluorescence microscopy. Created with BioRender.com.

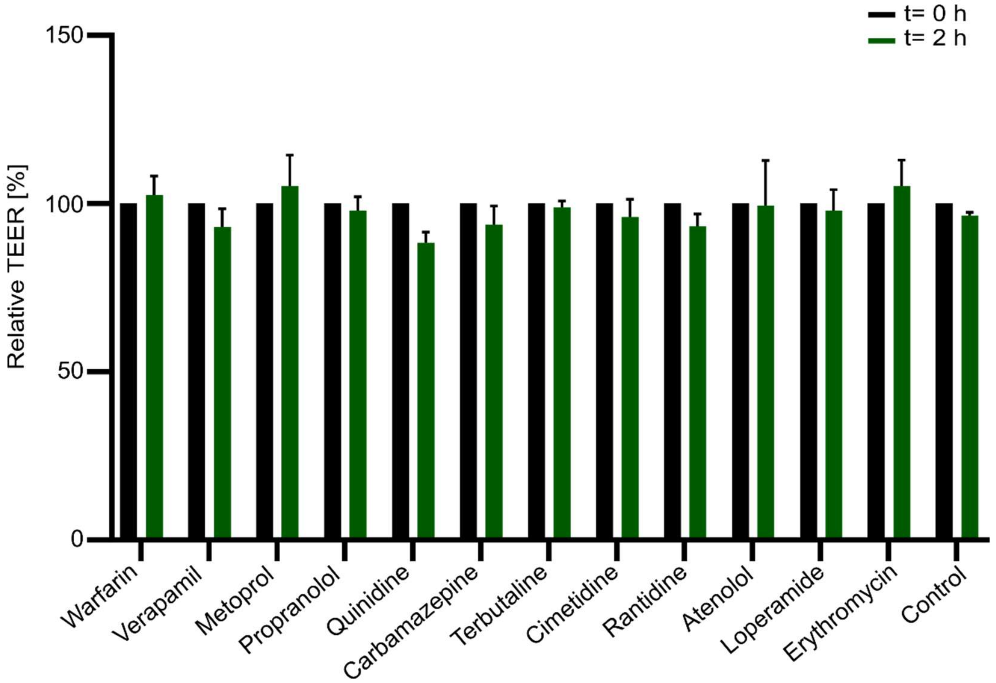

Supplementary Figure 4. Intact barrier function after 2 h of drug exposure on Transwells. The relative change in TEER was determined before the drug exposure (black) and after incubation with small molecule drugs for 2 h (green) using the EVOM2 system equipped with an EndOhm-6G cup. No significant barrier disruptions were detected after the drug exposure, indicating a sufficient barrier integrity during drug transport. Error bars represent the SEM of the TEER for each drug exposure measured for at least three biological replicates (n=3).

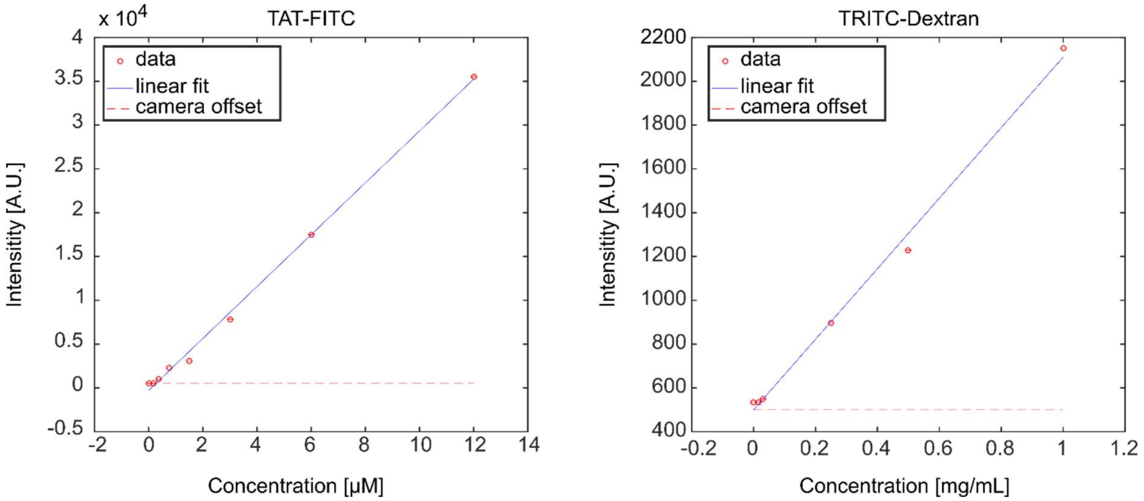

Supplementary Figure 5. Concentration curves of TAT and TRITC-Dextran for image-based quantification of TAT transport. The fluorescence signal of several concentrations of TAT (left) and TD (right) was measured for generating a calibration curve allowing the conversion of measured intensity values to the corresponding absolute concentration. The concentrations used for TAT were 0, 0.09, 0.187, 0.75, 1.5, 3, 6, 12 µM. For TD 0, 0.016, 0.03, 0.25, 0.5 and 1 mg mL^{-1} were added to empty chips and detected. Keeping all microscope settings identical the fluorescence intensity was measured in the ECM channel as described for the transport experiments in the main text and the individual data points acquired were fitted to a linear function.

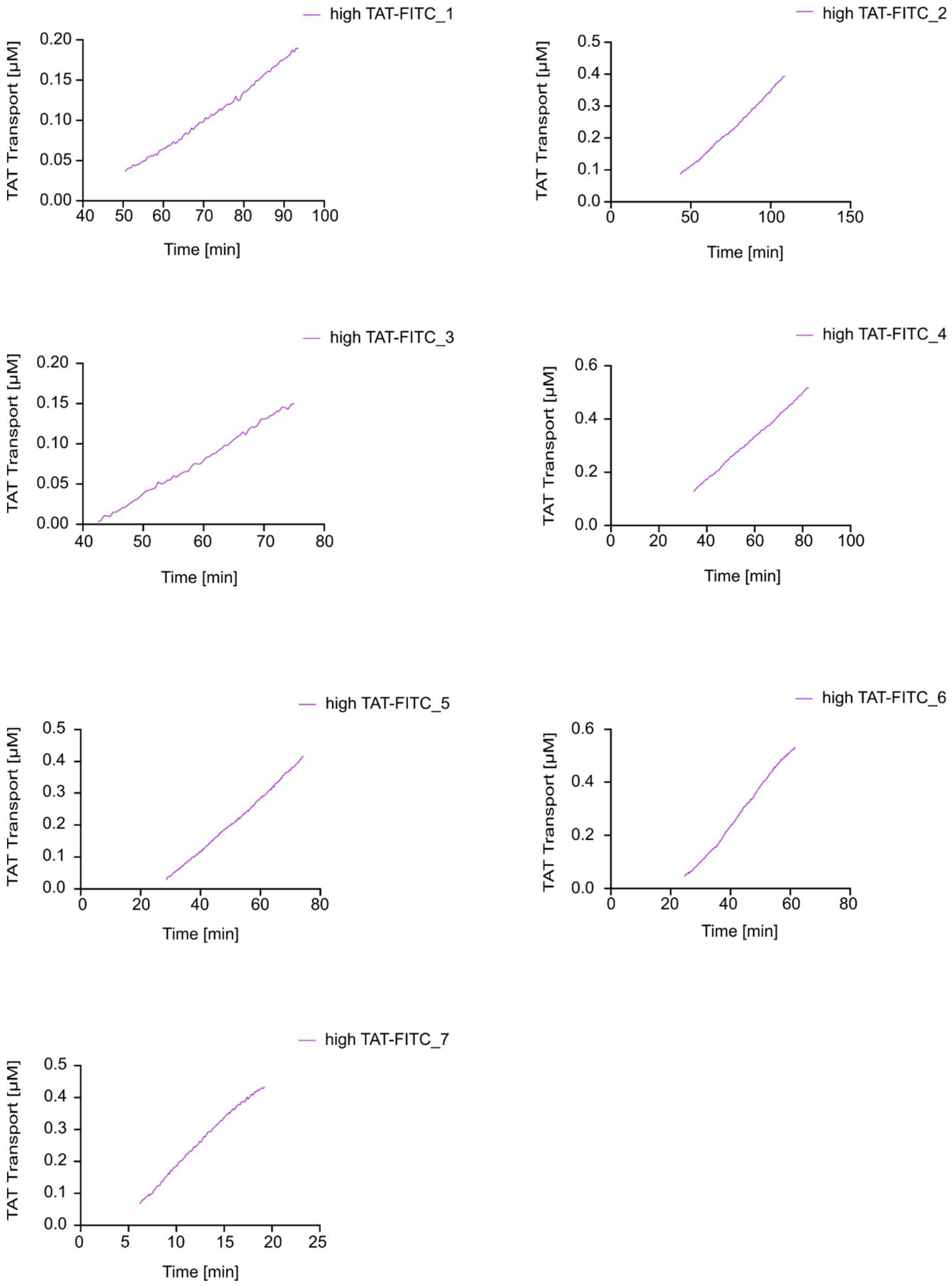

Supplementary Figure 6. Linear regime extracted from the kinetic profile of individual 24 µM TAT transport over time. The linear function of the selected area was determined and the curve's slope was used to compare the rate of transport between the applied concentrations of TAT. All kinetic profiles of TAT showed a similar linear regime. The acquired images of

the TAT (24 µM) were used to extract the fluorescent intensity profile and convert it into the actual fraction transported overtime using MATLAB for each individual replicate.

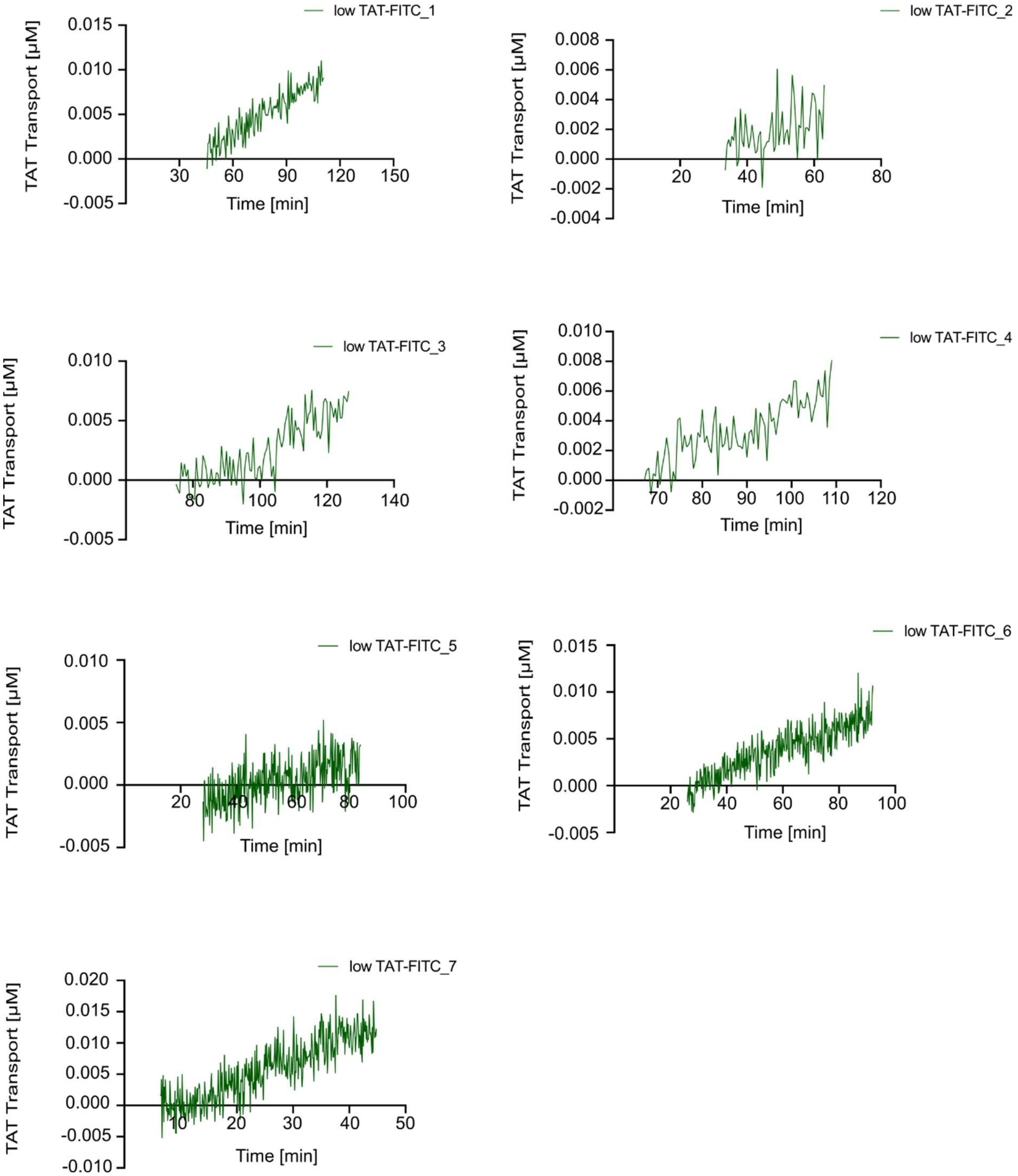

Supplementary Figure 7. Linear regime extracted from the kinetic profile of individual 2 µM TAT transport over time. The linear function of the selected area was determined and the slope factor was used to compare the rate of transport between the applied concentrations

of TAT. All kinetic profiles of TAT showed a similar linear regime. The acquired images of the TAT (2 μM) were used to extract the fluorescent intensity profile and convert it into the actual fraction transported over time using MATLAB for each individual replicate.

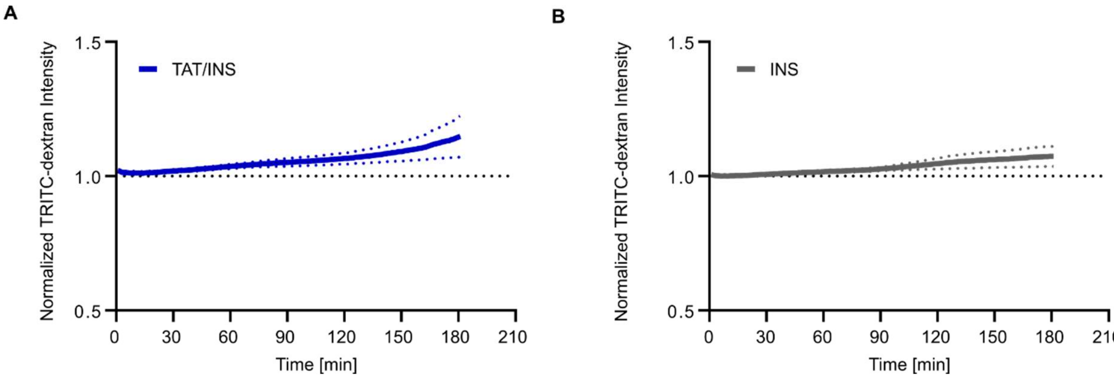

Supplementary Figure 8. Intact barrier integrity after TAT/INS exposure assed by simultaneous TRITC-dextran acquisition. Normalized TRITC-dextran intensity in coculture chips co-administered with TAT/INS (**A**) or with INS (**B**) over 180 min. Error bars represent the standard error of the mean, n=3.

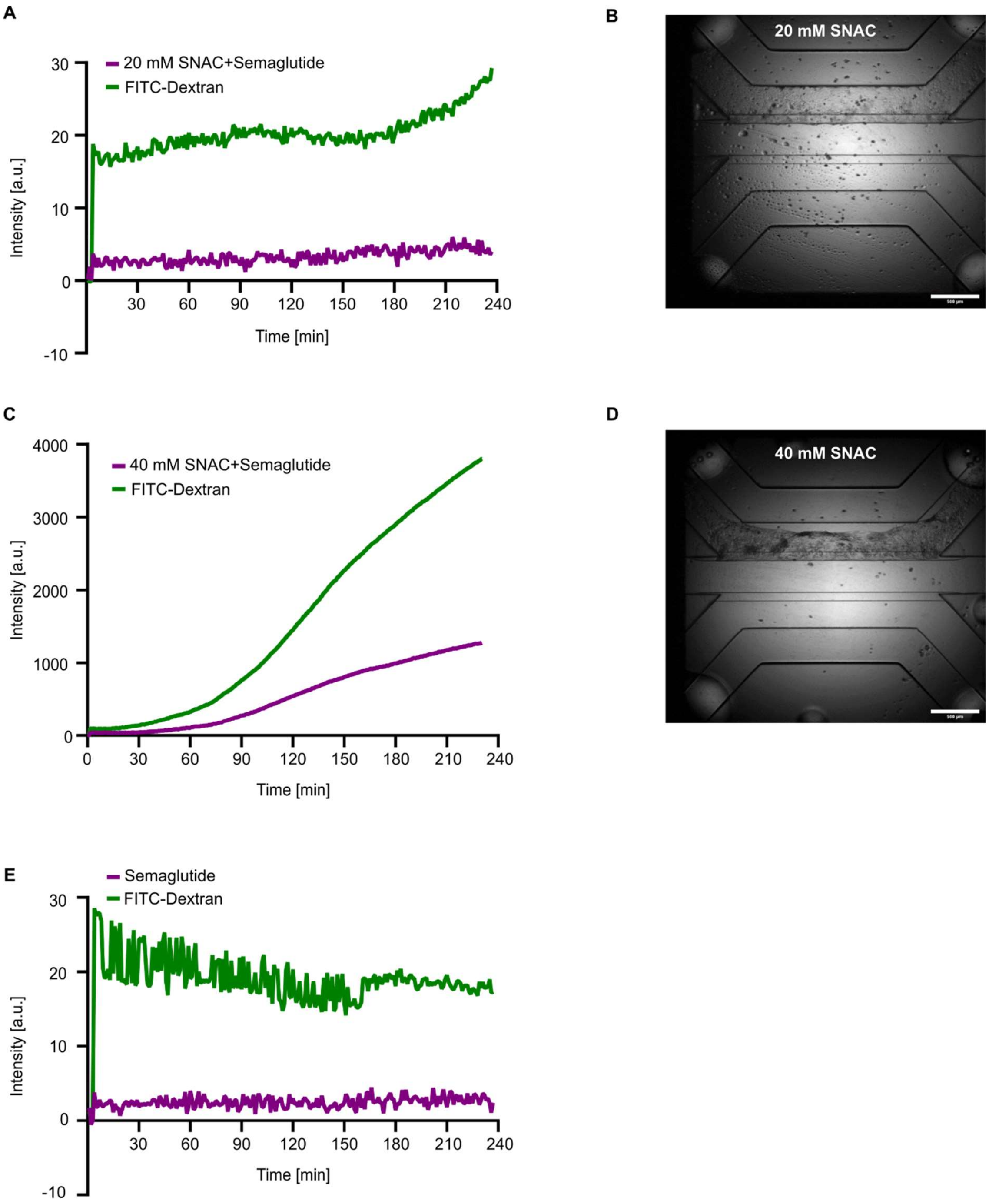

Supplementary Figure 9. Barrier integrity impairment as the rate limiting step for SNAC induced semaglutide transport. (**A**) Transport of semaglutide (purple) using 20 mM SNAC over 240 min, revealing the absence of increased semaglutide intensity over time. FITC-dextran (green) was used and monitored simultaneously as indicator for BI in all chips, showing no distinct transport of FITC-dextran (green) over time. (**B**) Bright field micrographs of chips after exposure for 240 min to 20 mM SNAC, illustrating an intact cell layer without signs of

SNAC induced toxicity. (**C**) Transport of semglutide (purple) over 240 min induced by 40 mM SNAC, showed after a lag phase of 30 min an clear increase in semaglutide fluorescence intensity measured in the ECM. In parallel to the detectable semaglutide signal, an increase of FITC-dextran (green) fluorescence was detected over time, accentuating the impairment of the barrier integrity. (**D**) Micrographs of coculture tubules show partial removal of cell layer after exposure to 40 mM SNAC, revealing a destabilization effect of SNAC on the cells. (**E**) As a negative control semaglutide alone was applied to coculture chips and the transport was measured over 240 min, demonstrating no significant change in fluorescence intensity of semaglutide (purple) and FITC-dextran (green). Scale bar is 500 µm. Data represent the mean of at least two biological replicates per condition.

Supplementary Table 1. HPLC gradient.

HPLC gradient	
Time [min.]	MeCN conc.
0	0%
1	0%
10	100%
11	100%
11.2	0%
14	0%

HPLC instrument: Nexera X2 SIL-30AC autosampler, Nexera X2 LC-30AD solvent pumps, Prominence CTO-20AC column oven, Prominence SPD-M20A diode array detector, DGU-20A5R degasser

Supplementary Table 2. Conditions for drug quantification by LC-MS.

	Capillary voltage	Nebulizer pressure	Dry gas flow	Dry gas temperature	End plate offset
Atenolol, Rantidine, Metoprolol, Propranolol, Verpamil, Warfarin, Loperamide	4,500 V	0.7 bar	3.0 L/min.	180 °C	-300 V
Cimetidine, Quinidine	1,000 V	0.5 bar	3.0 L/min.	180 °C	-800 V
Carbamazepine	4,500 V	1.5 bar	4.0 L/min.	180 °C	-400 V
Erythromycine	4,500 V	1.5 bar	4.0 L/min.	180 °C	-300 V
Terbutaline*	6,000 V	4.0 bar	9.0 L/min.	220 °C	-500 V

*The HPLC flow rate was reduced to 0.2 mL min^{-1} and a T-junction was inserted between the HPLC and the ion source to reduce the flow into the ion source even further (to approximately 0.1 mL min^{-1}).

Supplementary Table 3. **Barrier function determination after drug exposure using Lucifer Yellow (LY) in chips with mono- and coculture tubules and Transwells.** The leakage of LY was detected after 2 h of drug exposure in the individual platform. In all platforms and for all selected drug no significant LY-leakage was determined. The barrier maintained intact during the drug transport.

	Lucifer Yellow leakage (%)		
	Transwell	Chip<br>Caco-2	Chip<br>Caco-2 :HT29-MTX E12
Warfarin	0.02	0.03	0.01
Verapamil	0.03	0.04	0.01
Metoprolol	0.02	0.02	0.09
Propranolol	0.02	0.03	0.03
Quinidine	0.07	0.03	0.01
Carbamazepine	0.02	0.02	0.01
Terbutaline	0.02	0.02	0.01
Cimetidine	0.03	0.03	0.02
Rantidine	0.02	0.02	0.01
Atenolol	0.02	0.02	0.01
Loperamide	0.02	0.68	0.02
Erythromicin	0.03	0.04	0.01
Control	0.02	0.01	0.11

Supplementary Table 4. Comparison of apparent permeability in chips using mono-and coculture tubules and Transwell system of 12 model drugs.

Drug	Code	MW	TW* Caco-2 P_{app}**	Chip Caco-2 P_{app}**	Chip Caco-2:HT29-MTX E12 P_{app}**	BCS	FA***, in human %
Warfarin	1	308.33	243.41± 37.25	160.97± 40.49	300.39 ± 16.27	I	98 (Skolnik et al., 2010)
Verapamil	2	491.06	50.79± 5.16	33.53± 3.61	10.41 ± 1.75	I	95 (Skolnik et al., 2010)
Metoprolol	3	684.81	47.63± 5.44	17.67± 4.98	44.99 ± 1.05	I	95 (Youhanna and Lauschke, 2021)
Propranolol	4	295.8	101.68± 23.00	71.42± 5.08	86.05 ± 21.67	I	93 (Takenaka et al., 2016)
Quinidine	5	324.42	47.96± 5.71	16.61± 4.56	20.18 ± 3.36	I	80 (Skolnik et al., 2010)
Carbamazepine	6	236.27	204.43± 9.20	213.99± 28.88	140.04 ± 6.25	II	100 (Skolnik et al., 2010)
Terbutaline	7	274.32	1.85± 0.10	7.11± 0.43	5.09 ± 0.06	II	44 (Cheng Li et al., 2007)

Cimetidine	8	252.34	5.08± 1.39	9.79± 3.41	11.87 ± 3.41	III	62 (Takenaka et al., 2016)
Rantidine	9	350.86	3.22± 0.93	1.80± 0.27	2.29 ± 0.27	III	50 (Takenaka et al., 2016)
Atenolol	10	266.34	3.52± 0.55	3.41± 0.37	3.65 ± 0.45	III	50 (Skolnik et al., 2010)
Loperamide	11	513.5	58.60± 8.62	11.03± 1.62	2.75 ± 1.13	II	40 (Skolnik et al., 2010)
Erythromicin	12	733.93	1.20± 0.14	1.33± 0.29	0.99 ± 0.32	III	35 (Youhanna and Lauschke, 2021)

*TW=Transwells

***Papp* correspond to apparent permeability coefficient from A→B in × 10-6 cm/sec.

*** Fraction absorbed in human (FA %).

Supplementary Table 5. Direct statistical comparison of drug transport in Transwells, mono-and coculture tubules of 12 model drugs using an unpaired t-test.

Drug	Transport route	TW vs. Mono Significance	TW vs. Co Significance	Mono vs. Co Significance
Warfarin	Transcellular	ns	ns	*
Verapamil	Transcellular	ns	**	**
Metoprolol	Transcellular	*	ns	**
Propranolol	Transcellular	ns	ns	ns
Quinidine	Transcellular	*	*	ns
Carbamazepine	Transcellular	ns	**	ns
Terbutaline	Paracellular	**	****	ns
Cimetidine	Paracellular	ns	ns	ns
Rantidine	Paracellular	ns	ns	ns
Atenolol	Paracellular	ns	ns	ns
Loperamide	Transcellular	**	**	*
Erythromycin	Transcellular	ns	ns	ns

*In Supplementary Table 5 we assigned significance levels based on the premise: *$p< 0.05$ (*), $p< 0.01$ (**), $p<0.001$ (***) and $p<0.0001$ (****).*

References

Cheng Li, Tongtong Liu, Xiaoming Cui, Uss, A. S., and Cheng, K.-C. (2007). Development of In Vitro Pharmacokinetic Screens Using Caco-2, Human Hepatocyte, and Caco-2/Human Hepatocyte Hybrid Systems for the Prediction of Oral Bioavailability in Humans. *Journal of Biomolecular Screening* 12, 1084–1091. doi: 10.1177/1087057107308892.

Skolnik, S., Lin, X., Wang, J., Chen, X.-H., He, T., and Zhang, B. (2010). Towards Prediction of In Vivo Intestinal Absorption Using a 96-Well Caco-2 Assay. *Journal of Pharmaceutical Sciences* 99, 3246–3265. doi: 10.1002/jps.22080.

Takenaka, T., Harada, N., Kuze, J., Chiba, M., Iwao, T., and Matsunaga, T. (2016). Application of a Human Intestinal Epithelial Cell Monolayer to the Prediction of Oral Drug Absorption in Humans as a Superior Alternative to the Caco-2 Cell Monolayer. *Journal of Pharmaceutical Sciences* 105, 915–924. doi: 10.1016/j.xphs.2015.11.035.

Youhanna, S., and Lauschke, V. M. (2021). The Past, Present and Future of Intestinal In Vitro Cell Systems for Drug Absorption Studies. *Journal of Pharmaceutical Sciences* 110, 50–65. doi: 10.1016/j.xphs.2020.07.001.
